# A Concise Synthesis of a BODIPY-Labeled Tetrasaccharide Related to the Antitumor PI-88

**DOI:** 10.3390/molecules26102909

**Published:** 2021-05-14

**Authors:** Juan Ventura, Clara Uriel, Ana M. Gomez, Edurne Avellanal-Zaballa, Jorge Bañuelos, Inmaculada García-Moreno, Jose Cristobal Lopez

**Affiliations:** 1 Instituto de Química Orgánica General, IQOG-CSIC, Juan de la Cierva 3, 28006 Madrid, Spain; juan.ventura@csic.es (J.V.); clara.uriel@csic.es (C.U.); 2 Departamento de Química Física, Universidad del Pais Vasco-EHU, Apartado 644, 48080 Bilbao, Spain; eavellanal002@ehu.eus; 3 Instituto de Química-Física “Rocasolano”, IQFR-CSIC, Serrano 119, 28006 Madrid, Spain; i.garcia-moreno@iqfr.csic.es

**Keywords:** PI-88, glycosylation, 1,2-methyl orthoesters, BODIPY, fluorescent labeling

## Abstract

A convergent synthetic route to a tetrasaccharide related to PI-88, which allows the incorporation of a fluorescent BODIPY-label at the reducing-end, has been developed. The strategy, which features the use of 1,2-methyl orthoesters (MeOEs) as glycosyl donors, illustrates the usefulness of suitably-designed BODIPY dyes as glycosyl labels in synthetic strategies towards fluorescently-tagged oligosaccharides.

## 1. Introduction

**Figure 1:**
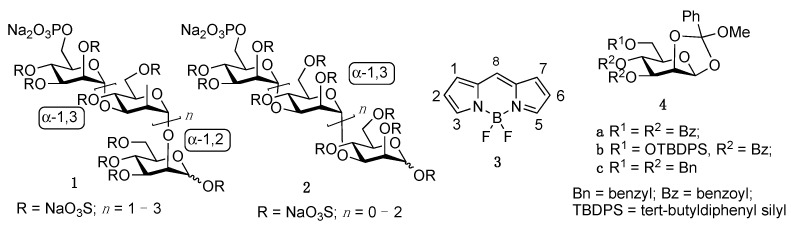
PI-88 (**1**,**2**), BODIPY (**3**), IUPAC numbering) and 1,2-methyl orthoester glycosyl donors (**4**) used in this study.

The recognition of the crucial role of heparanase enzyme, an endo-β-D-glucuronidase able to degrade heparan sulfate (HS) in the extracellular matrix and basement membranes, in a number of pathological processes, such as metastasis and angiogenesis, has triggered the development of heparanase inhibitors [1,2,3,4,5,6]. Among these inhibitors PI-88, a mixture of functionalized mannose oligosaccharides, i.e., **1, 2**, (Figure 1), that potently inhibited heparanase and in vitro angiogenesis, has been considered as a promising candidate and has received considerable attention [7]. On the other hand, fluorescent labeling of biomolecules has been recognized as a research topic of great significance, since such labeling facilitates the investigation of glycoconjugates and their interaction in biological systems at high sensitivity [8,9,10,11,12]. Among the types of fluorescent dyes commonly employed as tags, borondipyrromethene (BODIPY, 4, 4-difluoro-4-bora-3a, 4a-diaza-s-indacene) dyes, e.g., **3** (Figure 1) have excelled. Several reasons can be cited for this preference: their high fluorescent quantum yields (∅), excellent photochemical and chemical stabilities, and, arguably, the relatively facile modulation of their photophysical and/or chemical properties by means of synthetic postfunctionalization of their indacene core [13,14,15,16,17,18].

Based on these precedents, we thought it would be of interest to investigate the feasibility of a synthetic approach to BODIPY-labeled PI-88 saccharide components, where the fluorescent dye is incorporated from the beginning of the synthetic sequence. Additionally, we envisioned that the incorporation of the lipophilic BODIPY moiety at the reducing end of a PI-88 saccharide analogue would bring one additional advantage by facilitating the visualization and detection of the synthetic intermediates along the saccharide synthesis [19]. Additionally, in light of some reported literature precedents [20], the incorporation of the lipophilic BODIPY core to the saccharidic ensemble could lead to ameliorated biological activity in the ensuing saccharides. Thus, in this *Article*, we report a synthetic approach to a PI-88 tetrasaccharide analogue [21] featuring the use of 1,2-methyl orthoester (MeOE) glycosyl donors, i.e., **4** (Figure 1), in which a BODIPY-type fluorescent probe could be attached at the reducing end of the saccharides from the beginning of the synthesis.

## 2. Results

**Scheme 1:**
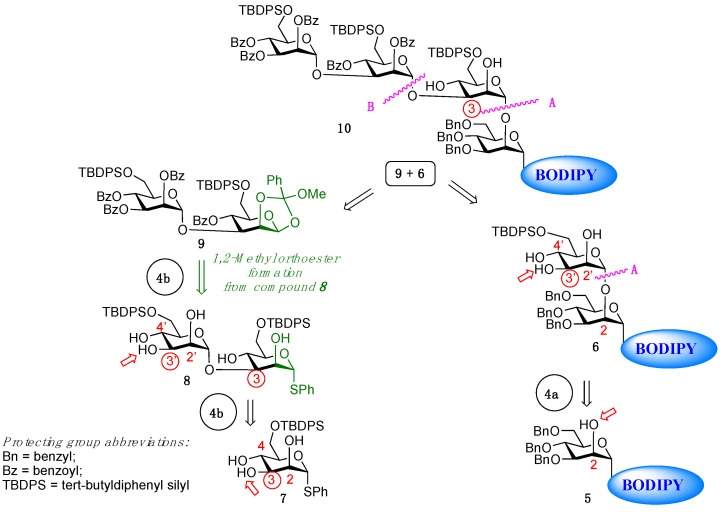
Retrosynthesis of PI-88 tetrasaccharide analogue **10**, involving the sole use of 1,2-methyl orthoester glycosyl donors, **4** and **9**.

Several synthetic approaches to analogues of tetra- and pentasaccharides **1** have already been reported [22,23,24,25,26,27,28]. In those approaches, different types glycosyl donors have been employed. We have been interested in the use of 1,2-methyl orthoesters (MeOEs) [29,30,31,32,33] as an inexpensive alternative to Fraser–Reid’s *n*-pentenyl orthoester glycosyl donors (NPOEs) [34,35,36]. The former derivatives were shown to display good regioselectivity, similar to that of NPOEs [31]. Accordingly, our convergent 1 + 1 + 2 approach to BODIPY-tagged tetramannan, **10** (Scheme 1) was based on our previous studies on the selective mono-glycosylation at position *O*-3 in mannopyranose substrates possessing a 2,3,4 triol moiety, i.e., **6**, **7** (Scheme 1), with MeOE glycosyl donors, **4a**, **4b**, and **9 [31]**. Thus, our strategy involved two glycosidic disconnections **A** and **B** (Scheme 1). Disconnection **A** (Scheme 1), leading to BODIPY-disaccharide **6**, was envisioned by glycosylation of a benzylated mannopyranoside **5**, exposing only one hydroxyl group (*O*-2) for the glycosyl coupling, with MeOE donor **4a**. On the other hand, disconnection **B** (Scheme 1) was imagined by regioselective mono-glycosylation of triol **6**, at position *O*-3’, according to precedents from our research group [31], by a disaccharide MeOE donor, **9**.

**Scheme 2:**
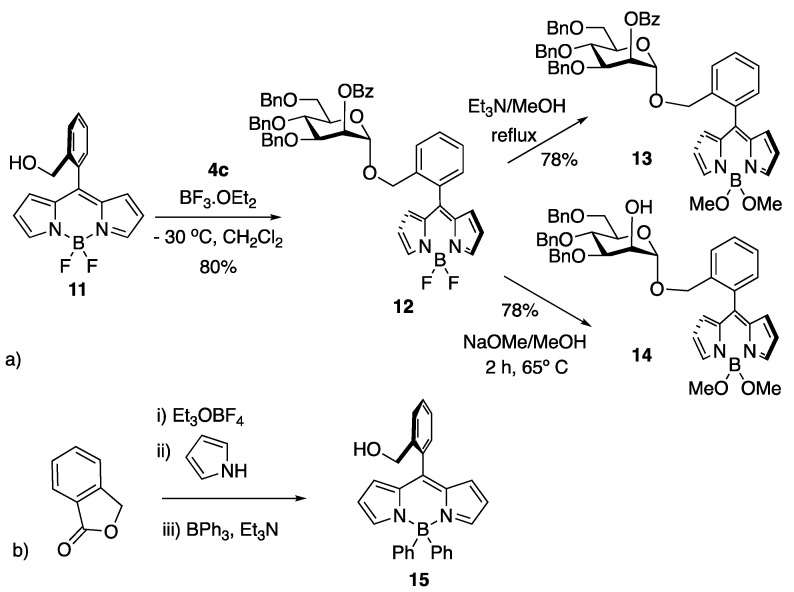
(**a**) Glycosylation of BODIPY **11** with MeOE **4c** and saponification attempts; (**b**) one-pot synthesis of 4,4-diphenyl BODIPY **15**.

The synthetic route started with the glycosylation of BODIPY **11 [37]**, readily available through a one-pot transformation using phthalide and pyrrole as the starting materials, followed by in situ coordination with BF_3_.OEt_2_ [38], with methyl orthoester **4c**, to yield BODIPY glycoside **12** (80% yield, Scheme 2a). However, attempted saponification of the 2-*O*-Bz substituent in **12**, by treatment with Et_3_N or NaOMe in methanol, resulted in the production of the undesired B(OMe)_2_-BODIPY derivatives **13** and **14**, where the fluorine atoms were replaced by methoxy groups (Scheme 2a). These derivatives, although also fluorescent [39], proved to be labile under the acidic conditions required in the next glycosylation events. We then turned our attention to borondiphenyl BODIPY **15**, a more chemically robust yet fluorescent BODIPY analogue [40]. Access to **15** was also affected by slightly modifying our one-pot procedure [38], from phthalide and pyrrole, by simply replacing BF_3_.OEt_2_ by B(Ph)_3_ in the borondipyrromethene ring closing reaction (Scheme 2b) [41].

**Scheme 3:**
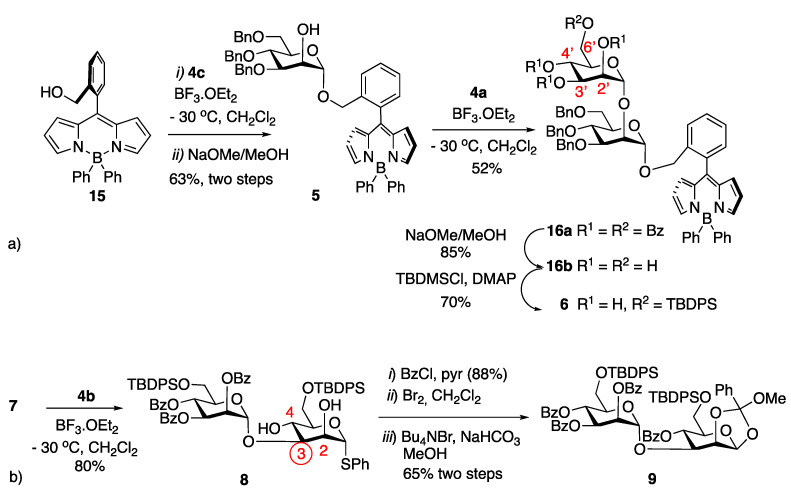
(**a**) Synthesis of BODIPY disaccharide acceptor **6**; (**b**) access to MeOE-disaccharide donor **9**, from thioglycoside **7**, by regioselective glycosylation with MeOE **4b** and subsequent orthoester formation on the ensuing disaccharidic thioglycoside intermediate **8**.

The synthetic routes to the two fragments in the convergent approach to PI-88 tetrasaccharide analogue **10**, glycosyl acceptor **6** and glycosyl donor **9**, are depicted in Scheme 3a,b. Thus, glycosylation of hydroxymethyl BODIPY **15** with benzylated MeOE **4c**, followed by saponification (NaOMe/MeOH), led to BODIPY-mannopyranoside **5** (Scheme 3a). The latter was then glycosylated with tri-*O*-benzoyl MeOE **4a**, to yield BODIPY disaccharide **16a** in moderate yield (52%). Next, protecting group manipulations in compound **16a**, including de-*O*-benzoylation leading to tetraol **16b**, and selective monosilylation at the primary hydroxyl group at O-6’ in the latter [terbutyldiphenylsilyl chloride, 4-dimethylaminopyridine (DMAP), in dimethyl formamide (DMF)] produced 2’,3’,4’-triol **6**, (Scheme 3a).

On the other hand, the route to MeOE-disaccharide donor **9** started from phenyl thiomannopyranoside **7** (Scheme 3b). Accordingly, regioselective mono-glycosylation at *O*-3 of 2,3,4-triol **7** with MeOE **4b,** based on precedents from our research group [31,42], yielded phenyl 1-thiomannopyranoside **8** in good yield (Scheme 3b). To conclude, a three-step sequence from **8**, including perbenzoylation, anomeric bromination, and orthoester formation from an intermediate glycosyl bromide according to Wei et al. [43], allowed its conversion to MeOE disaccharide donor **9** (Scheme 3b).

**Scheme 4:**
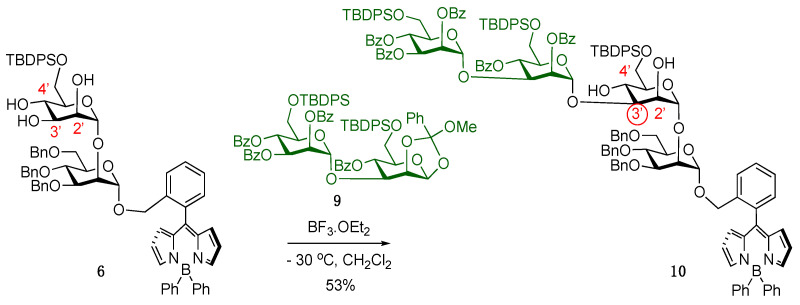
Regioselective glycosylation of triol **6** with MeOE-disaccharide **9** leading to tetrasaccharide **10**.

Finally, glycosyl acceptor **6**, containing a mannopyranoside triol unit, was regioselectively mono-glycosylated at *O*-3’with MeOE disaccharide **9** [44], to yield PI-88 tetrasaccharide precursor analogue **10**, in 53% yield (Scheme 4). An alternative glycosylation of **6** with a MeOE monosaccharide, e.g., **4a** or **4b**, rather than with a disaccharide, i.e., **9**, in our hands consistently led to low yields of the trisaccharide analogue.

**Figure 2:**
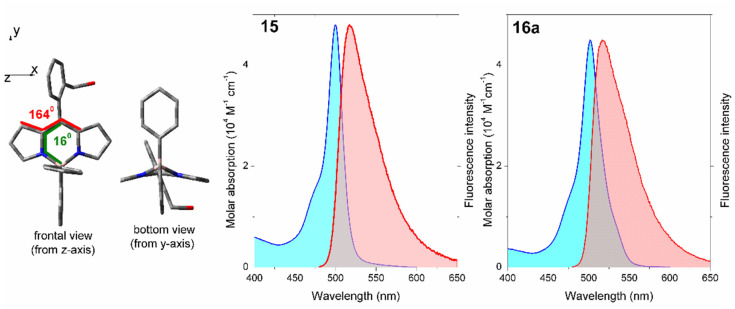
Absorption (in blue) and normalized fluorescence (in red) spectra of BODIPY **15** and its glycosylated derivative **16a** in diluted solution of ethyl acetate. The spectral profiles of **10** fully resemble those of **16a**. The excited state optimized geometry of **15** in two different views is also enclosed with key dihedral angles to show the bending of the chromophore.

To impel the advanced applications of the new glycoprobes, we analyzed the photonic behavior of BODIPY **15** and its saccharide derivatives **16a** and **10**, under low (photophysical properties) and high (laser properties) irradiation regimes. The replacement of the fluorine atoms at the boron bridge, as in **11 [38]**, by phenyl groups, as in **15** [45], had low impact on the spectral properties of BODIPY (Figure 2) but induced both a decrease of the emission efficiency (Table 1) and a biexponential character of the fluorescent lifetime (Table S1), regardless of the environmental properties. The free motion of these phenyl rings chelating the boron atom reduced the planarity of the dipyrrin core (computed bending angles in the dipyrrin core up to 16° in the excited state, Figure 2), increasing the internal conversion processes with a deleterious effect on the fluorescence signal. The labelling of a disaccharide or tetrasaccharide with BODIPY **15**, as in compound **16a** and **10**, respectively, widened the absorption spectrum, while the spectral profile of fluorescence matched that of its precursor (Figure 2). It is noteworthy that BODIPYs **10** and **16a** displayed a brighter fluorescence with longer lifetimes than their non-glycosylated counterpart **15** in all tested media and regardless of the number of saccharide units appended (Table 1 and Table S1). Thus, glycosylation of the *ortho*-hydroxymethyl group of the C-8-aryl residue led to a more rigid and compact molecular structure, e.g., **16a** and **10**, owing to the higher steric hindrance imposed by the bulky disaccharide. In fact, the structural arrangement of the C-8-benzyl residue in **16a** was nearly orthogonal (twisting dihedral angle computed in the ground state of 75° in **15** vs. 85° in **16a**), reducing the internal conversion pathways associated to conformational freedom. Consequently, BODIPY-saccharides **16a** and **10** behaved as efficient and stable fluorescent glycoprobes even under laser irradiation conditions, exhibiting a lasing efficiency up to 38% in the green spectral region (540 nm, Table 1) with high photostability, since their laser emission remained at the initial level even after 70,000 pump pulses. This good tolerance to intense and prolonged irradiation is a highly desirable property for fluorescent labels to provide long-lasting bioimages. Therefore, from a photonic point of view, the *ortho*- position of 8-phenyl BODIPYs is highlighted as a suitable grafting position to tag (oligo)saccharides, even resulting in an amelioration of the photonic performance of the original labeling dye.

## 3. Conclusions

In summary, we developed a convergent, efficient, synthetic strategy to BODIPY-labeled PI-88 tetrasaccharide components (**10**) [46], which serves to illustrate the scope and usefulness of MeOEs as glycosyl donors. The inclusion of the BODIPY-tag from the beginning of the synthesis facilitates the visual recognition (thin-layer chromatography, TLC) of the labeled-saccharide acceptor and the glycosylated products therefrom, among the rest of the non-fluorescent side-products arising from side-reactions of the MeOE glycosyl donor [36]. This feature becomes particularly appealing when excess amounts of glycosyl donors are required to lead the glycosylation to completion. On the other hand, the chemically stable 4, 4’-diphenyl BODIPY derivative (**15**), used as a tag, displayed good fluorescent properties and photostability under strong and prolonged irradiation, and was also able to withstand all reaction conditions employed in the synthetic sequence leading to **10** [20]. Our results also indicate that the incorporation of carbohydrate subunits at the *ortho*-hydroxymethyl group of the C-8-aryl substituent has a beneficial effect on the, already, good photophysical features of the BODIPY dye.

## 4. Materials and Methods

### 4.1. General Information

The solvents and reagents used in the transformations included in the manuscript were obtained from commercial sources. In the glycosylation experiments, the adventitious water content was removed by repeated evaporation of the sample with toluene. The temperature at which the reactions were carried out will be mentioned unless room temperature was used. The glycosylation reactions were carried out in dried flasks fitted with rubber septa under an argon atmosphere.

A 5.0 M stock solution of triethyloxonium tetrafluoroborate, employed in the preparation of the BODIPYs, was prepared by dissolving 25 g (0.131 mmol) of the salt in 26.3 mL of anhydrous methylene chloride.

Anhydrous MgSO_4_ was used to dry organic solutions during workup. Evaporation of the solvents was performed using a rotary evaporator (Buchi, Flawil, Switzerland). Flash column chromatography was used to purify or separate the samples. Thin-layer chromatography (TLC) was conducted on Kieselgel 60 F254. Spots corresponding to BODIPY-containing molecules were spotted under visible light. TLCs were then inspected under UV irradiation (254 nm) followed by charring with a solution of 20% aqueous H_2_SO_4_ (200 mL) in AcOH (800 mL). ^1^H and ^13^C-NMR spectra were recorded in CDCl_3_ at 300, 400, or 500 MHz and 75, 101, or 126 MHz, respectively. Chemical shifts are expressed in parts per million (δ scale) downfield from tetramethylsilane and are referenced to residual protium in the NMR solvent (CHCl_3_: δ 7.25 ppm, CD_3_OD: δ 4.870 ppm). Coupling constants (*J*) are given in Hz. All presented ^13^C-NMR spectra are proton decoupled. Mass spectra were recorded by direct injection with an Accurate Mass Q-TOF LC/MS spectrometer (Agilent Technologies, Santa Clara, CA, USA) equipped with an electrospray ion source in positive mode.

### 4.2. General Procedures

#### 4.2.1. General Procedure for Glycosylation. Procedure A

A previously dried mixture of a glycosyl donor and glycosyl acceptor was dissolved in anhydrous dichloromethane (≈3 mL/0.1 mmol). Previously dried (200 °C, one night) 4Å molecular sieves were added to the mixture. The reaction was cooled to −30 °C, and then BF_3_.OEt_2_ (3.0 equiv) was added. After 5–10 min, the reaction mixture was diluted with dichloromethane and the ensuing solution washed with saturated aqueous NaHCO_3_ solution. The organic layer was dried over Na_2_SO_4_, filtered, and evaporated under vacuum. The resulting crude mixture was purified by chromatography on silica gel (eluent: hexane-ethyl acetate mixtures).

#### 4.2.2. General Procedure for Debenzoylation. Procedure B

The corresponding compound was dissolved in methanol (25 mL/ mmol) and triethylamine (6 mL/ mmol) was added to the resulting solution. The reaction mixture was refluxed overnight, the solvents evaporated, and the ensuing residue concentrated. Purification by flash chromatography was carried out using hexane-ethyl acetate mixtures, as eluent.

#### 4.2.3. General Procedure for Silylation. Procedure C

The corresponding compound was dissolved in dry DMF (20 mL/mmol), and to this solution imidazole (4 equiv.) was added. After stirring for 5–10 min in an ice bath, under argon, terbutyldiphenylsilyl chloride (1.2 equiv.) and a small amount of dimethylaminopiridine DMAP were added. The ice bath was removed, and the reaction mixture was left with stirring at room temperature for 24 h. The reaction mixture was then diluted with ethyl acetate and extracted with a saturated aqueous NaHCO_3_ solution and brine. The combined organic solutions were dried over anhydrous MgSO_4_ and evaporated under vacuum. The resulting crude mixture was purified by chromatography on silica gel (eluent: hexane-ethyl acetate mixtures).

#### 4.2.4. General Procedure for Characterization of Polyol Derivatives as Peracetates. Procedure D

The corresponding polyol was dissolved in pyridine (1 mL/0.1 mmol substrate) and acetic anhydride (0.5 mL/mmol substrate) was then added. The reaction mixture was stirred at room temperature (normally 24 h). After completion of the reaction (t.l.c.), the solvent was evaporated, and the resulting crude mixture was purified by chromatography on silica gel (eluent: hexane-ethyl acetate mixtures).

### 4.3. Synthesis

#### 4.3.1. BF_2_-Bodipy **11**

This compound was prepared according to our previously described method [38], from phthalide and pyrrole.

#### 4.3.2. Mannopyranosyl BODIPY **12**

This compound was prepared by glycosylation of BODIPY **11** (26 mg, 0.088 mmol) with orthoester **4c** (100 mg, 0.178 mmol), according to the general procedure for glycosylation, procedure A. Flash chromatography (hexane- ethyl acetate; 85:15) yielded BODIPY-mannoside **12** (64 mg, 80%). ^1^H-NMR (500 MHz, CDCl_3_) δ 8.05–7.16 (m, 24H), 6.68 (dd, *J* = 12.4, 4.2 Hz, 2H), 6.47 (dd, *J* = 4.2, 1.8 Hz, 1H), 6.33 (dd, *J* = 4.3, 1.8 Hz, 1H), 5.40 (dd, *J* = 3.1, 1.9 Hz, 1H), 4.86 (d, *J* = 1.9 Hz, 1H), 4.79 (d, *J* = 10.7 Hz, 1H), 4.72–4.65 (m, 3H), 4.52–4.42 (m, 4H), 4.37 (d, *J* = 11.8 Hz, 1H), 4.01 (t, *J* = 9.6 Hz, 1H), 3.88–3.75 (m, 3H), 3.58 (dd, *J* = 10.9, 1.9 Hz, 1H), 3.50–3.44 (m, 1H) ^13^C-NMR (125 MHz, CDCl_3_) δ 165.7, 145.3, 145.2, 144.8, 138.7, 138.6, 138.2, 135.7, 135.4, 133.2, 132.9, 132.6, 131.2, 131.0 (×2), 130.2, 130.1 (×2), 130.0, 128.9, 128.5, 128.4 (×5), 128.2 (×2), 127.9, 127.7 (×2), 127.6 (×2), 119.2, 118.8, 97.5, 78.1, 75.3, 74.1, 73.5, 72.0, 71.6, 68.8 (×3), 68.3, 67.3, 51.1, 29.8; ^19^F-NMR (376 MHz, CDCl_3_) δ −145.4 (q, *J*_B–F_ = 28.9 Hz), −145.4 (q, *J*_B–F_ = 28.9 Hz); HRMS (ESI-TOF): calc for C_50_H_49_BF_2_N_3_O_7_ [M + NH_4_]^+^; 852.37407 found 852.36969.

#### 4.3.3. Mannopyranosyl BODIPY **13**

This compound was obtained when applying the general procedure for debenzoylation, procedure B, to BODIPY-mannoside **12** (60 mg, 0.087 mmol) followed by flash chromatography (hexane- ethyl acetate; 6:4). Compound **13** (56 mg, 78%): ^1^H-NMR (300 MHz, CDCl_3_) δ 8.04–7.12 (m, 24H), 6.66 (ddd, *J* = 10.4, 4.2, 1.1 Hz, 2H), 6.48 (ddd, *J* = 4.2, 1.8, 0.7 Hz, 1H), 6.43 (ddd, *J* = 4.2, 1.8, 0.7 Hz, 1H), 5.42 (dd, *J* = 3.1, 2.0 Hz, 1H), 4.87–4.77 (m, 2H), 4.74–4.60 (m, 3H), 4.51–4.36 (m, 4H), 4.04 (t, *J* = 9.5 Hz, 1H), 3.98–3.88 (m, 1H), 3.78 (dd, *J* = 10.6, 3.5 Hz, 1H), 3.66–3.53 (m, 2H) 3.12 (s, 3H), 3.03 (s, 3H); ^19^F-NMR (376 MHz, CDCl_3_): showed not peaks at all; HRMS (ESI-TOF): calc for C_52_H_51_BN_2_NaO_9_ [M + Na]^+^; 881.35682 found 881.35690.

#### 4.3.4. Mannopyranosyl BODIPY **14**

Treatment of compound **12** (80 mg, 0.096 mmoles) with sodium methoxide in MeOH, at 65 °C for 1 h resulted in the consumption of the starting material. Then, addition of solid NH_4_Cl and stirring for 15 min followed by filtration and solvent evaporation provided a residue that was purified by chromatography on silica gel (hexane-ethyl acetate; 3:7) to give dimethoxyboron-derivative **14** (56 mg, 78%). ^1^H-NMR (300 MHz, CDCl_3_) δ 7.88–7.03 (19H), 6.68 (dd, *J* = 4.2, 1.3 Hz, 1H), 6.60 (dd, *J* = 4.2, 1.3 Hz, 1H), 6.42 (dt, *J* = 4.3, 1.8 Hz, 1H), 4.74 (d, *J* = 10.8 Hz, 1H), 4.59–4.35 (m, 9H), 3.78–3.50 (m, 6H), 3.38–3.33 (m, 1H), 3.20 (s, 3H), 3.06 (s, 3H); ^13^C-NMR (125 MHz, CDCl_3_) δ 152.8, 144.9, 144.5, 136.7, 136.6, 135.7, 133.7, 133.6, 130.4, 130.0, 129.8, 129.7, 129.4, 128.5 (×2), 128.4 (×2), 128.3 (×2), 128.1 (×2), 128.0 (×2), 127.8 (×2), 127.7 (×2), 118.5, 118.1, 100.6, 79.9, 75.2, 74.1, 73.6, 71.7, 71.4, 69.1, 68.5, 68.3, 67.3, 50.3, 49.8 HRMS (ESI-TOF): calc for C_45_H_47_BN_2_NaO_8_ [M + Na]^+^; 777.33254 found 777.33546.

#### 4.3.5. B(Ph)_2_-BODIPY **15**

This compound was prepared by treatment of a toluene solution of a dipyrromethane intermediate (obtained by reaction of phthalide with pyrrole) (44 mg, 0.198 mmol), with triphenylborane (48 mg, 0.198 mmol). The mixture, under argon, was refluxed for 24 h. Then, the solvent was evaporated, and the residue dissolved in dichloromethane (10 mL), to which 10 mL of a 1M solution of NaOH were added. The ensuing mixture was kept with stirring for one night. The organic layer was separated and dried over MgSO4, filtered, and evaporated. The residue was purified by for chromatography on silica gel (hexane-ethyl acetate; 8:2) to afford compound **15** (38 mg, 46%). ^1^H-NMR (400 MHz, CDCl_3_) δ 7.64–6.97 (m, 14H), 6.75 (d, *J* = 4.2 Hz, 2H), 6.55–6.39 (m, 2H), 4.35 (s, 2H) ^13^C-NMR (100 MHz, CDCl_3_) δ 145.5 (×2), 145.1, 139.7, 135.5, 133.8 (×2), 132.5, 131.7 (×2), 129.8, 129.7, 128.7 (×2), 128.2, 127.6 (×3), 127.1, 126.7, 126.2, 118.0 (×2), 62.9 HRMS (ESI-TOF): calc for C_28_H_24_BN_2_O_8_ [M + H]^+^; 415.19812 found 415.19815.

#### 4.3.6. Mannopyranosyl BODIPY **5**

This compound was prepared by glycosylation of BODIPY **15** (73 mg, 0.176 mmol) with MeOE **4c** (150 mg, 0.264 mmol), according to the general procedure for glycosylation, procedure A. After standard work-up, purification by flash chromatography (hexane- ethyl acetate; 9:1) furnished a mixture of the 2-*O*-benzoyl derivative of compound **5** (102 mg, 72%) and “rearranged” methyl mannopyranoside (^1^HNMR). This mixture was dissolved in MeOH, under argon, and treated with NaOMe/MeOH at room temperature for 24 h. When t.l.c. showed disappearance of the fluorescent starting material, solid NH4Cl was added. The resulting solution was kept with stirring for 15 min, filtered, and the solvent was evaporated. Purification by flash chromatography (hexane-ethyl acetate; 7:3) allowed the isolation of BODIPY-mannoside **5** (92 mg, 87%). ^1^H-NMR (500 MHz, CDCl_3_) δ 7.61 (s, 1H), 7.52 (s, 1H) 7.47–7.09 (m, 29H), 6.70 (dd, *J* = 26.9, 4.3 Hz, 2H), 6.43–6.34 (m, 2H), 4.74 (d, *J* = 10.7 Hz, 1H), 4.60 (d, *J* = 12.2 Hz, 1H), 4.53–4.39 (m, 4H), 4.37 (bs, 2H), 4.28 (d, *J* = 11.6 Hz, 1H), 3.73 (t, *J* = 9.5 Hz, 1H), 3.64–3.46 (m, 4H), 3.39–3.35 (m, 1H); ^13^C-NMR (125 MHz, CDCl_3_) δ 145.3 (×2), 145.2, 138.4, 138.1, 136.0, 135.6, 134.9, 133.8, 133.5 (×2), 132.2 (×3), 130.2, 129.6, 129.5, 129.2, 129.1, 128.6 (×2), 128.5 (×2), 128.4 (×3), 128.1 (×2), 128.0 (×2), 127.9, 127.8 (×2), 127.7 (×2), 127.6 (×2), 126.6, 126.3, 117.8, 117.6, 98.6, 80.2, 75.3, 74.1, 73.5 (×2), 71.7, 71.3, 68.8, 67.7, 67.3 HRMS (ESI-TOF): calc for C_55_H55BN_3_O_6_ [M + NH_4_]^+^; 846.41876 found 846.41891.

#### 4.3.7. BODIPY Disaccharide **16a**

This compound was prepared by glycosylation of **5** (70 mg, 0.083 mmol) with MeOE **4a** (57 mg, 0.100 mmol), according to the general procedure for glycosylation, procedure A. Purification by flash chromatography (hexane-ethyl acetate; 7:3) afforded compound **16a** (62 mg, 52%). ^1^H-NMR (400 MHz, CDCl_3_) δ 8.08–7.05 (m, 49 H), 6.69 (ddd, *J* = 12.0, 4.3, 1.2 Hz, 2H), 6.39 (ddd, *J* = 19.1, 4.3, 1.8 Hz, 2H), 6.11 (t, *J* = 10.1 Hz, 1H), 5.93 (dd, *J* = 10.1, 3.2 Hz, 1H), 5.87 (dd, *J* = 3.2, 1.9 Hz, 1H), 5.17 (d, *J* = 2.0 Hz, 1H), 4.84 (d, *J* = 10.9 Hz, 1H), 4.80 (d, *J* = 1.9 Hz, 1H), 4.66 (d, *J* = 12.3 Hz, 1H), 4.60–4.39 (m, 7H), 4.42 (d, *J* = 12.1 Hz, 1H), 4.33 (dd, *J* = 12.3, 3.6 Hz, 1H), 4.24 (d, *J* = 12.0 Hz, 1H), 4.00 (t, *J* = 9.6 Hz, 1H), 3.79 (d, *J* = 2.4 Hz, 1H), 3.75–3.63 (m, 2H), 3.61–3.52 (m, 2H); ^13^C-NMR (125 MHz, CDCl_3_) δ 166.2, 165.6, 165.3, 165.1, 145.5, 145.4, 144.9, 138.5, 138.5, 138.3, 136.0, 135.2 (×2), 133.5 (×2), 133.2 (×3), 133.1 (×2), 132.5 (×2), 132.4, 130.3, 130.2, 130.0 (×6),129.9 (×2), 129.6 (×2), 129.4 (×2), 129.1, 128.9, 128.7 (×2), 128.6 (×2), 128.5 (×4), 128.4 (×5), 128.3 (×2), 127.9 (×3), 127.8, 127.7 (×2), 127.6 (×2), 127.5 (×3), 126.4 (×2) (65 signals for aromatic carbons) 117.8, 117.7, 99.4, 97.8, 79.5, 75.9, 75.2, 74.4, 73.3 (×2), 72.2, 72.0, 70.2, 70.0, 69.1, 68.7, 66.8, 66.7, 62.6 HRMS (ESI-TOF): calc for C_89_H_77_BKN_2_O_15_ [M + K]^+^; 1447.51131 found 1447.51484 (M + K)^+^.

#### 4.3.8. BODIPY-Disaccharide Tetraol **16b**

This compound was prepared according to the general procedure for debenzoylation, procedure B, from compound **16a** (60 mg, 0.087 mmol). Purification by flash chromatography (hexane-ethyl acetate; 2:8) afforded fluorescent disaccharide **16b** (40 mg, quantitative yield). ^1^H-NMR (500 MHz, CDCl_3_) δ 7.56–7.11 (m, 29H), 6.69–6.66 (m,2H), 6.37 (ddd, *J* = 12.6, 4.3, 1.8 Hz, 2H), 4.93 (bs, 1H), 4.74 (d, *J* = 10.8 Hz, 1H), 4.67 (bs, 1H) 4.56–4.51 (m, 2H), 4.42–4.36 (m, 4H), 4.25 (d, *J* = 12.3 Hz, 1H), 3.98 (bs, 1H), 3.87–3.77 (m, 5H), 3.69–3.64 (m,3H), 3.59–3.52 (m, 4H), 3.44–3.37 (m, 4H) ^13^C-NMR (125 MHz, CDCl_3_) δ 145.5, 145.3, 145.0, 138.5 (×2), 138.4, 136.0, 135.2, 135.1, 133.7, 133.4, 133.2 (×2), 133.1, 132.9, 132.5 (×2), 130.3, 130.2, 129.6, 129.1, 128.8 (22), 128.5 (×5), 128.4 (×2), 128.0 (×2), 127.8 (×3), 127.7 (×3), 127.6 (×4), 126.5, 126.4, 117.9 (×2), 101.5, 97.6, 79.5, 75.3, 75.2, 74.4, 73.4, 72.5, 72.1, 72.0, 71.6, 70.9, 68.8, 66.6, 61.6; API-ES positive mode: [M + Na]^+^ = 1031.3.

#### 4.3.9. BODIPY-Disaccharide Silylated Triol **6**

The general procedure for silylation, procedure C, was applied to tetraol **16b** (56 mg, 0.056 mmol), although this time pyridine, rather than DMF, was used as solvent. Purification by flash chromatography (hexane-ethyl acetate; 6:4) yielded triol **6** (48 mg, 69%). ^1^H-NMR (400 MHz, CDCl_3_) δ 7.72–7.10 (m, 39H), 6.64 (ddd, *J* = 5.6, 4.3, 1.2 Hz, 2H), 6.34 (ddd, *J* = 12.6, 4.3, 1.8 Hz, 2H), 4.97 (d, *J* = 1.7 Hz, 1H), 4.76 (d, *J* = 10.8 Hz, 1H), 4.65–4.59 (m, 2H), 4.54–4.38 (m, 4H), 4.36 (bs 2H), 4.18 (d, *J* = 12.2 Hz, 1H), 3.99 (bs, 1H), 3.87–3.57 (m, 13H), 3.45–3.40 (m, 2H),1.04 (s, 9H) ^13^C-NMR (100 MHz, CDCl_3_) δ 145.5, 145.4, 145.1, 138.8, 138.7, 138.5, 136.1, 135.8, 135.2, 133.4, 133.2, 133.1, 133.0, 132.7, 130.2, 129.5, 129.3, 128.6, 128.5, 128.1, 128.0, 127.8, 127.7, 127.6, 127.5, 126.5 (48 aromatic carbons), 118.0, 117.9, 101.2, 98.1, 79.9, 75.3, 74.6, 73.5 (×2), 72.3, 72.1 (×2), 71.5 (×2), 71.4, 70.5 (×2), 70.1, 69.1, 66.6, 65.1, 53.7, 27.1 (×3), 19.5; API-ES, positive mode: [M + Na]^+^ = 1269.30; HRMS (ESI-TOF): calc for C_156_H_155_BN_2_O_26_Si_3_ [M + Na]^+^; 2591.02041 found 2591.01683.

#### 4.3.10. Thioglycosyl Disaccharide **8**

According to the general procedure for glycosylation, procedure A, thioglycoside **7** (50 mg, 0.098 mmol) was glycosylated with orthoester **4b** (146 mg, 0.196 mmol) in CH_2_Cl_2_ (3 mL). After 5 min, the reaction was quenched, the organic extract concentrated, and the resulting residue chromatographed over silica gel flash column (hexane-ethyl acetate; 9:1) to give diol **8** (65 mg, 58%). For the sake of characterization, and according to the general procedure for acetylation, procedure D, the corresponding peracetyl derivative **8-Acet** was prepared (68 mg, quantitative yield). [α]_D_^21^: −39.3°, (*c* 0.8, CHCl_3_) ^1^H-NMR (400 MHz, CDCl_3_) δ 8.18–7.10 (m, 40H), 6.35 (t, *J* = 10.2 Hz, 1H), 5.71 (dd, *J* = 10.3, 3.3 Hz, 1H), 5.53–5.45 (m, 4H), 5.32 (d, *J* = 1.8 Hz, 1H), 4.38–4.34 (m, 1H), 4.25–4.20 (m, 2H), 3.92–3.80 (m, 2H), 3.78–3.61 (m, 2H), 2.20 (s, 3H), 2.12 (s, 3H), 1.07 (s, 9H), 1.06 (s, 9H) ^13^C-NMR (100 MHz, CDCl_3_) δ 170.5, 170.1, 165.8, 165.6, 165.5, 136.0 (×4), 135.9 (×2), 135.7 (×2), 133.9, 133.7, 133.5, 133.4, 133.3, 133.2, 133.0, 131.9 (×2), 130.2 (×2), 130.0 (×4), 129.9 (×2), 129.8, 129.7 (×2), 129.5 (×2), 129.3 (×2), 128.8 (×2), 128.6 (×2), 128.5 (×2), 127.9 (×4), 127.8 (×2), 99.4, 86.3, 75.6, 73.3, 72.9, 72.2, 71.2, 70.3, 68.2, 66.2, 63.2, 61.9, 26.95 (×3), 26.86 (×3), 21.7, 21.0, 19.5 (×2); API-ES, positive mode:1324.5 [M + NH_4_]^+^.

#### 4.3.11. 1,2-Methyl Orthoester Disaccharide **9**

Benzoylation (BzCl, pyridine) of compound **8** (498 mg, 0.41 mmol), followed by purification by flash chromatography (hexane-ethyl acetate; 85:15) provided the corresponding benzoylated disaccharide intermediate **8-Bzl** (450 mg, 88%). A portion of this compound (298 mg, 0.208 mmol) was dissolved in CH_2_Cl_2_ (3 mL), and cooled to 0 ºC, in the darkness, then bromine (15 μL, 0.314 mmol) was added. When the starting material had disappeared (t.l.c.) the reaction mixture was washed with 10% aqueous sodium thiosulfate solution containing sodium bicarbonate saturated aqueous solution, and water. The organic extract was dried over Na_2_SO_4_, filtered, and concentrated. Without further purification, the residue was dissolved in acetonitrile (1 mL) to which solution, methanol (84 μL), tetrabutylammonium bromide (Bu_4_NBr) (47 mg), sodium bicarbonate (35 mg), and molecular sieves 4Å (previously dried) were added. The resulting reaction mixture was stirred for one night, the molecular sieves were then filtered, and the solvent concentrated. The ensuing residue was purified by flash chromatography (hexane-ethyl acetate; 9:1, containing 1% of triethylamine ) to afford the 1,2-methyl orthoester disaccharide **9** (182.6 mg, 65% (two steps)) [α]_D_^21^: −89.2 (*c* 1.2, CHCl_3_) ^1^H-NMR (400 MHz, CDCl_3_) δ 8.05–7.09 (m, 45H), 6.20 (t, *J* = 10.1 Hz, 1H), 5.84 (dd, *J* = 10.2, 3.3 Hz, 1H), 5.62 (dd, *J* = 9.8, 8.7 Hz, 1H), 5.50 (d, *J* = 3.0 Hz, 1H), 5.44 (dd, *J* = 3.4, 1.8 Hz, 1H), 5.27 (d, *J* = 1.8 Hz, 1H), 4.84 (dd, *J* = 4.1, 3.0 Hz, 1H), 4.50 (d, *J* = 10.1 Hz, 1H), 4.23 (d, *J* = 9.8 Hz, 1H), 3.95–3.88 (m, 2H), 3.77–3.62 (m, 3H), 2.96 (s, 3H), 1.06 (s, 9H), 0.91 (s, 9H).^13^C-NMR (100 MHz, CDCl_3_) δ 165.5, 165.3 (×2), 165.2, 136.2, 136.0 (×2), 135.8 (×6), 133.5, 133.4, 133.3, 133.2, 133.1, 130.1 (×4), 130.0 (×2), 129.9 (×3), 129.8 (×2), 129.7 (×3), 129.6, 129.5, 129.4, 128.7 (×2), 128.6 (×2), 128.5 (×2), 128.4 (×4), 127.8 (×3), 127.7 (×4), 126.9 (×2), 122.9, 100.1, 98.1, 78.6, 77.9, 75.5, 72.2, 71.0, 70.5, 68.2, 66.6, 63.7, 62.4, 51.5, 26.9 (×3), 26.9 (×3), 19.5, 19.3 HRMS (ESI-TOF): calc for C_80_H_84_NO_16_Si_2_ [M + NH_4_]^+^ 1370.5323; found 1370.5323 [M + NH_4_]^+^.

#### 4.3.12. BODIPY-Tetrasaccharide **10**

BODIP disaccharide **6** (21 mg, 0.017 mmol) was glycosylated with glycosyl donor **9** (vide infra) (45 mg, 0.034 mmol), according to the general procedure for glycosylation, procedure A. Purification by flash chromatography (hexane-ethyl acetate; 8:2) yielded tetrasaccharide **10** (23 mg, 53%). ^1^H-NMR (500 MHz, CDCl_3_) δ 8.05–6.98 (m, 84H), 6.62 (td, *J* = 4.3, 1.3 Hz, 2H), 6.31 (dd, *J* = 4.3, 1.8 Hz, 1H), 6.28 (dd, *J* = 4.3, 1.8 Hz, 1H), 6.21 (t, *J* = 10.1 Hz, 1H), 5.66 (d, *J* = 8.7 Hz, 1H), 5.59–5.52 (m, 2H), 5.40 (bs, 1H) 5.37 (m, 1H), 5.31 (bs, 1H), 5.20 (bs, 1H), 4.71–4.69 (m, 2H), 4.62–4.45 (m, 6H), 4.39–4.28 (m, 5H), 4.21–4.03 (m, 4H), 3.99–3.68 (m, 12H), 3.64–3.56 (m, 2H), 3.49–3.35 (m, 4H), 1.02 (s, 9H), 0.99 (s, 9H), 0.95 (s, 9H) ^13^C-NMR (125 MHz, CDCl_3_) δ 165.7, 165.5, 165.2, 165.0 (×2), 145.4, 145.3, 144.9, 138.6, 138.6, 138.4, 138.0, 136.2, 135.9 (×4), 135.8 (×3), 135.7 (×3), 135.6 (×4), 135.1, 133.6, 133.5, 133.4, 133.3, 133.2, 133.1(×3), 133.0, 132.9, 132.7 (×2), 132.6 (×2), 132.3, 130.2 (×2), 130.1 (×2), 130.0 (×2), 129.9, 129.8 (×6), 129.7 (×2), 129.5 (×2), 129.4 (×2), 129.2 (×3), 128.8, 128.7, 128.5 (×4), 128.4 (×11), 128.2, 128.0, 127.9, 127.8, 127.7 (×5), 127.6, 127.4 (×4), 127.2, 126.5, 126.4, 125.4, 117.9 (×2), 101.1, 99.1, 98.2, 98.0, 81.5, 80.1, 76.2, 75.1, 74.6, 73.4 (×2), 72.9, 72.3 (×2), 72.2, 72.0, 71.9 (×2), 71.8, 70.6, 70.4, 69.5, 69.1, 68.3, 66.6, 66.2, 65.8, 64.4, 63.8, 61.5, 53.6, 27.0 (×3), 26.8 (×3), 26.7 (×3), 19.5, 19.3, 19.0; HRMS (ESI-TOF): calc for C_156_H_155_BN_2_O_26_Si_3_ [M + Na]^+^; 2591.02041 found 2591.01683.

## Figures and Tables

**Table 1:** Photophysical ^1^ and laser ^2^ properties of BODIPY **15**, and glycosylated derivatives **16a** and **10**, in ethyl acetate. For the sake of comparison, the corresponding photophysical data of the F-BODIPY counterpart (in ethanol) have been added. For additional photophysical data, see Table S1.

Compound	λ_ab_ (nm)	ε_max_	λ_fl_ (nm)	∅	<τ> (ns)	λ_la_ (nm)	Eff (%)
**11**	499.5	6.4	513.0	0.74	6.45	540	60
**15**	497.0	4.8	511.5	0.22	2.83	533	18
**16a**	500.0	4.5	516.0	0.31	3.19	545	38
**10**	500.5	4.1	515.0	0.31	3.57	547	36

^1^ Registered under a soft irradiation regime; dye concentration: 2 μM. Absorption (λ_ab_) and fluorescence (λ_fl_) wavelength, molar absorption (ε_max_) (10^4^ M^−1^ cm^−1^), fluorescence quantum yield (∅), and amplitude-average lifetime (<τ>). ^2^ Recorded under a hard irradiation regime; dye concentration 2 mM. Peak wavelength for the laser emission (λ_la_) and efficiency (Eff (%)) defined as the ratio between the energy of the laser output and the pump energy incident on the cell surface.

## Data Availability

Data sharing is not applicable to this article.

## Supplementary Materials

The following are available online, Phtophysical data (Table S1). Experimental conditions for photophysical properties, and quantum mechanical calculations. ^1^H- and ^13^C-NMR of all compounds.

## Sample Availability

No samples of the compounds are available from the authors.
