# Supplementary material for: A Concise Synthesis of a BODIPY-Labeled Tetrasaccharide Related to the Antitumor PI-88
Source: Molecules. 2021 May 14;26(10):2909. doi: 10.3390/molecules26102909 (PMC8156587; doi:10.3390/molecules26102909)
Supplement: Supplementary file 1 [file molecules-26-02909-s001.zip › molecules-1203481-supplementary.pdf]

A concise synthesis of a BODIPY-labeled tetrasaccharide related to the antitumor PI-88

Juan Ventura [1], Clara Uriel [1], Ana M. Gómez [1,*], Edurne Avellanal-Zaballa, [2] Jorge Bañuelos, [2,*] Inmaculada García-Moreno [3] and J. Cristóbal López [1,*]

[1] *Instituto de Química Orgánica General,* IQOG-CSIC, *Juan de la Cierva 3, 28006, Madrid, Spain.;*

[2] *Departamento de Química Física. Universidad del Pais Vasco-EHU, Apartado 644, 48080, Bilbao, Spain.*

[3] *Instituto de Química-Física "Rocasolano", IQFR-CSIC, Serrano 119, 28006, Madrid, Spain.*

* Correspondence: ana.gomez@csic.es (AMG); jorge.banuelos@ehu.eus (JB); jc.lopez@csic.es (JCL)

Supplementary Material

Copies of ^{1} H, ^{13}C, HSQC, ^{19}F spectra 2

Experimental: Photophysical and quantum mechanical properties 28

Laser experiments 29

Table S1. *Photophysical properties of dye* **15** *and its glycosylated derivatives* **10** *and* **16a** *(bearing a tetrasaccharide and a disaccharide respectively) in diluted solutions of different solvents* 30

Copies of [1] H, ^{13}C, HSQC, ^{19}F spectra

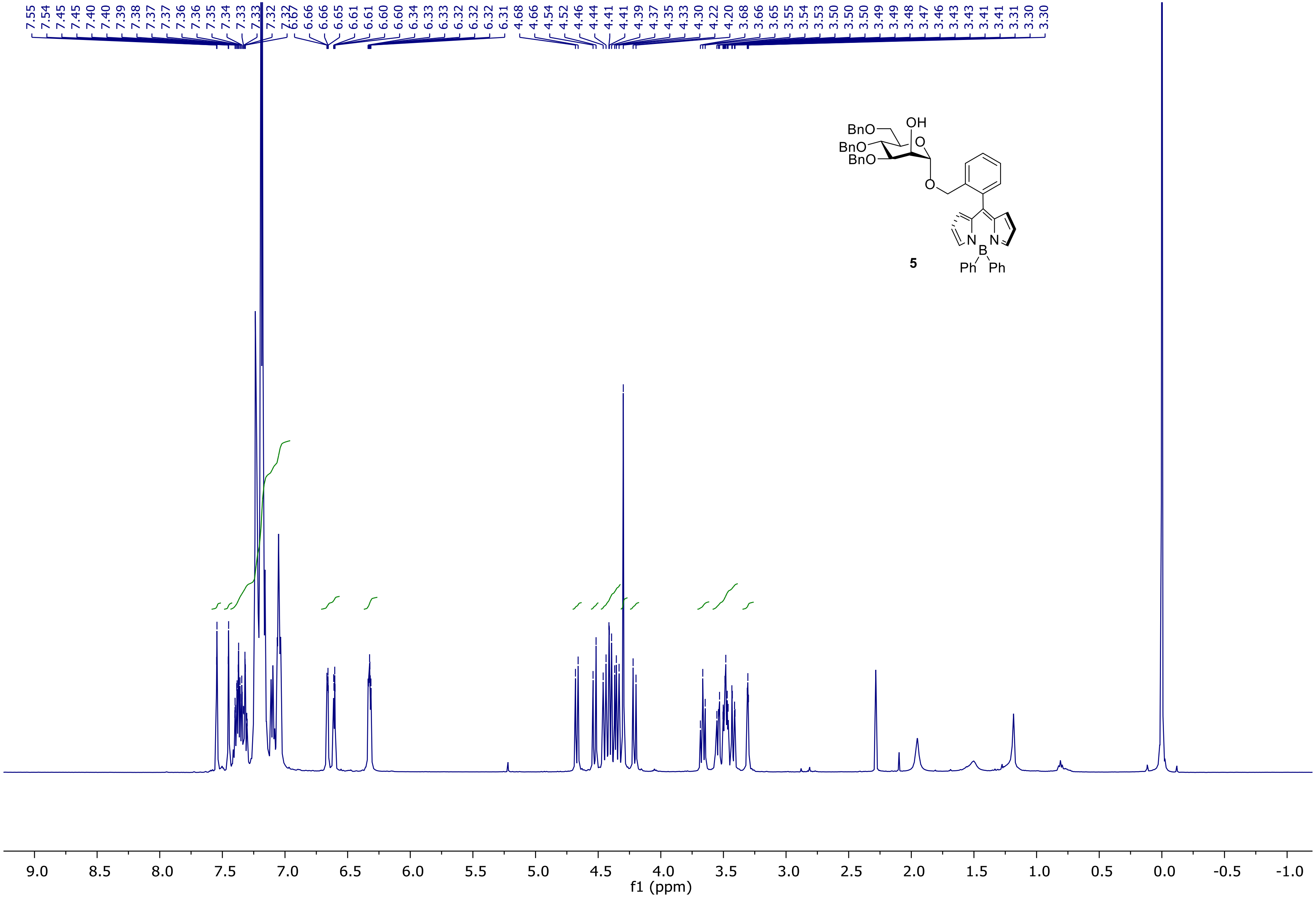

1H NMR (500 MHz, CDCl3)

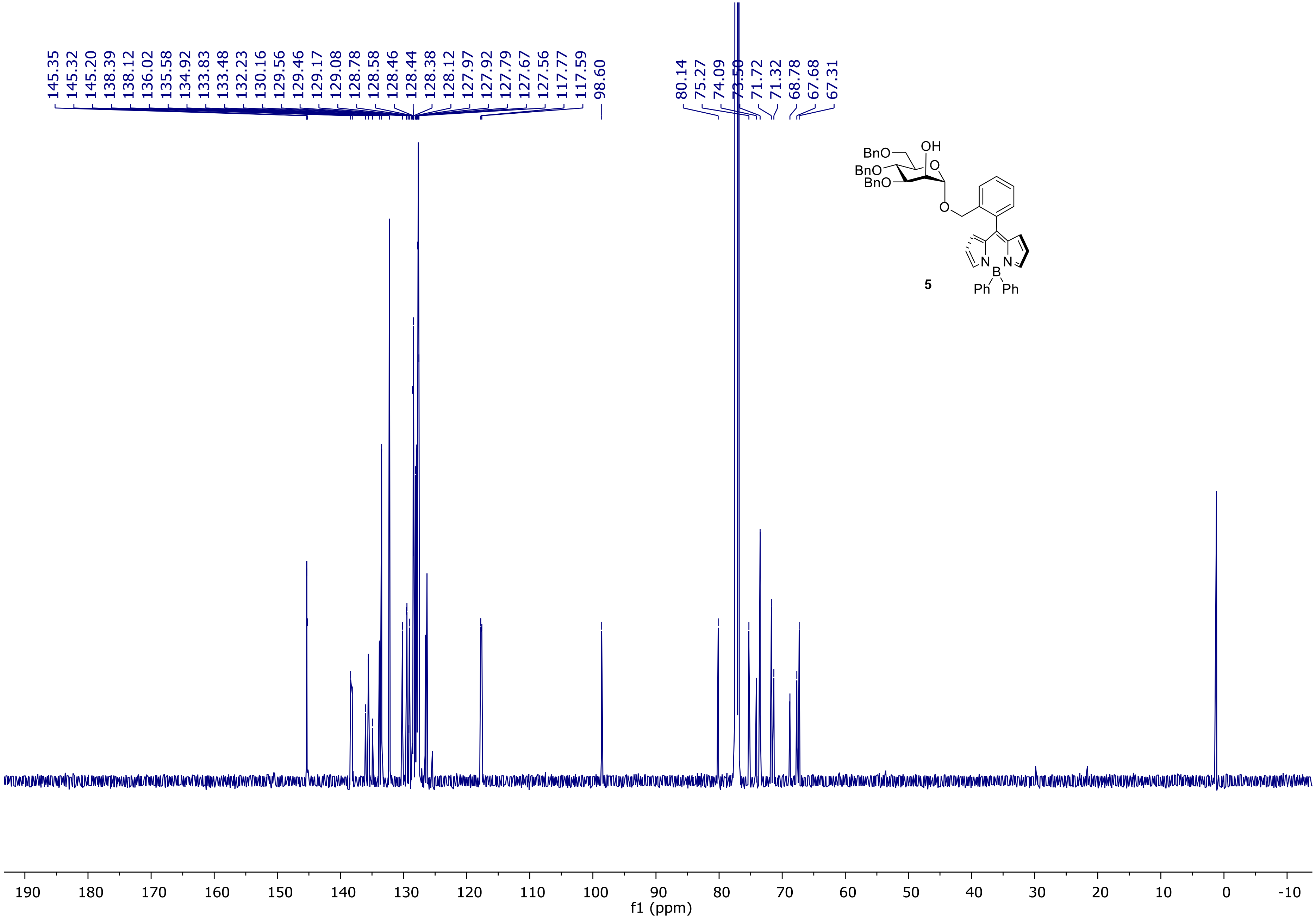

5

13C NMR (125 MHz, CDCl3)

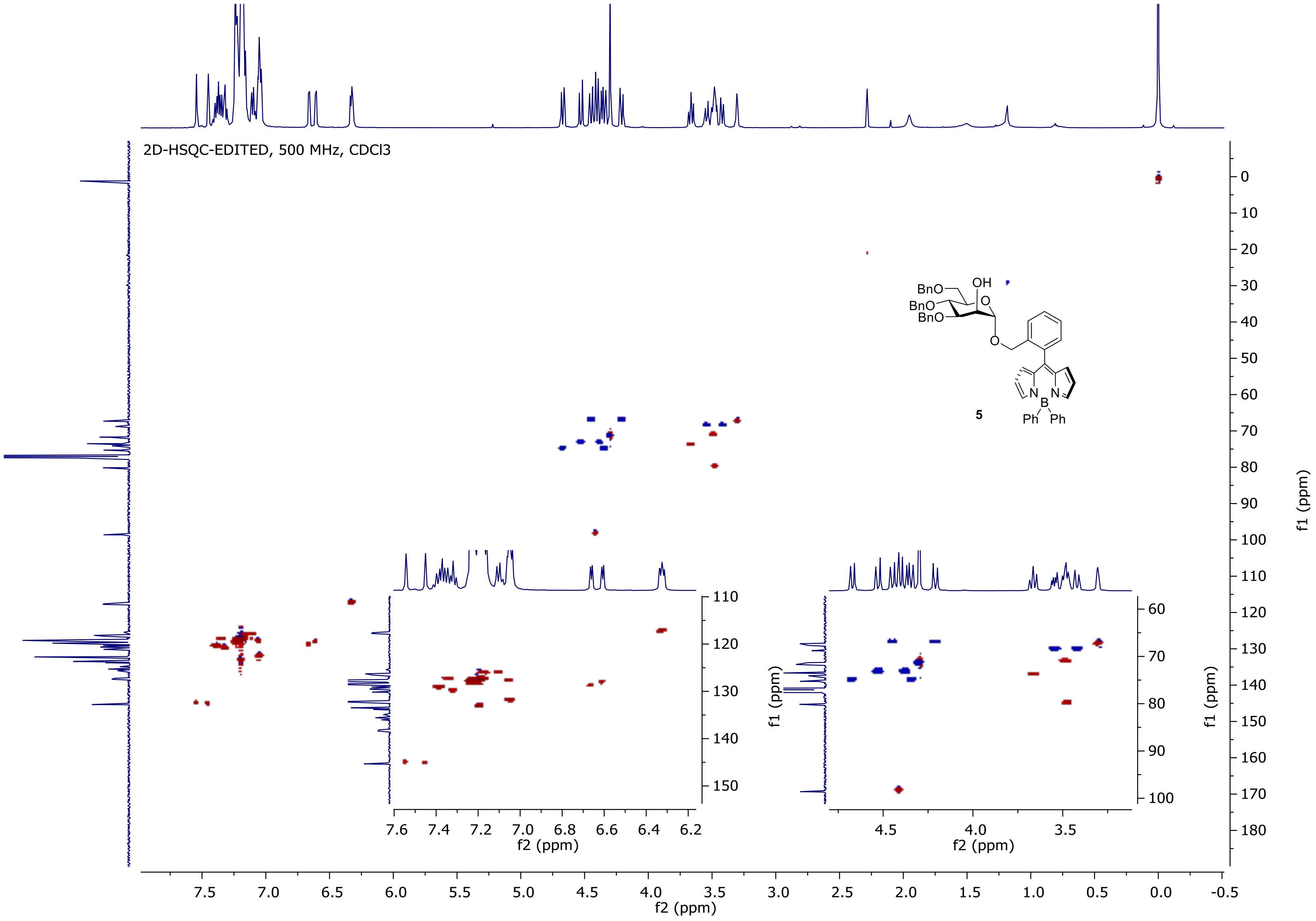
2D-HSQC-EDITED, 500 MHz, CDCl3
BnO
OH
O
BnO
BnO
O
N
B
N
Ph
Ph
5
f1 (ppm)
f2 (ppm)

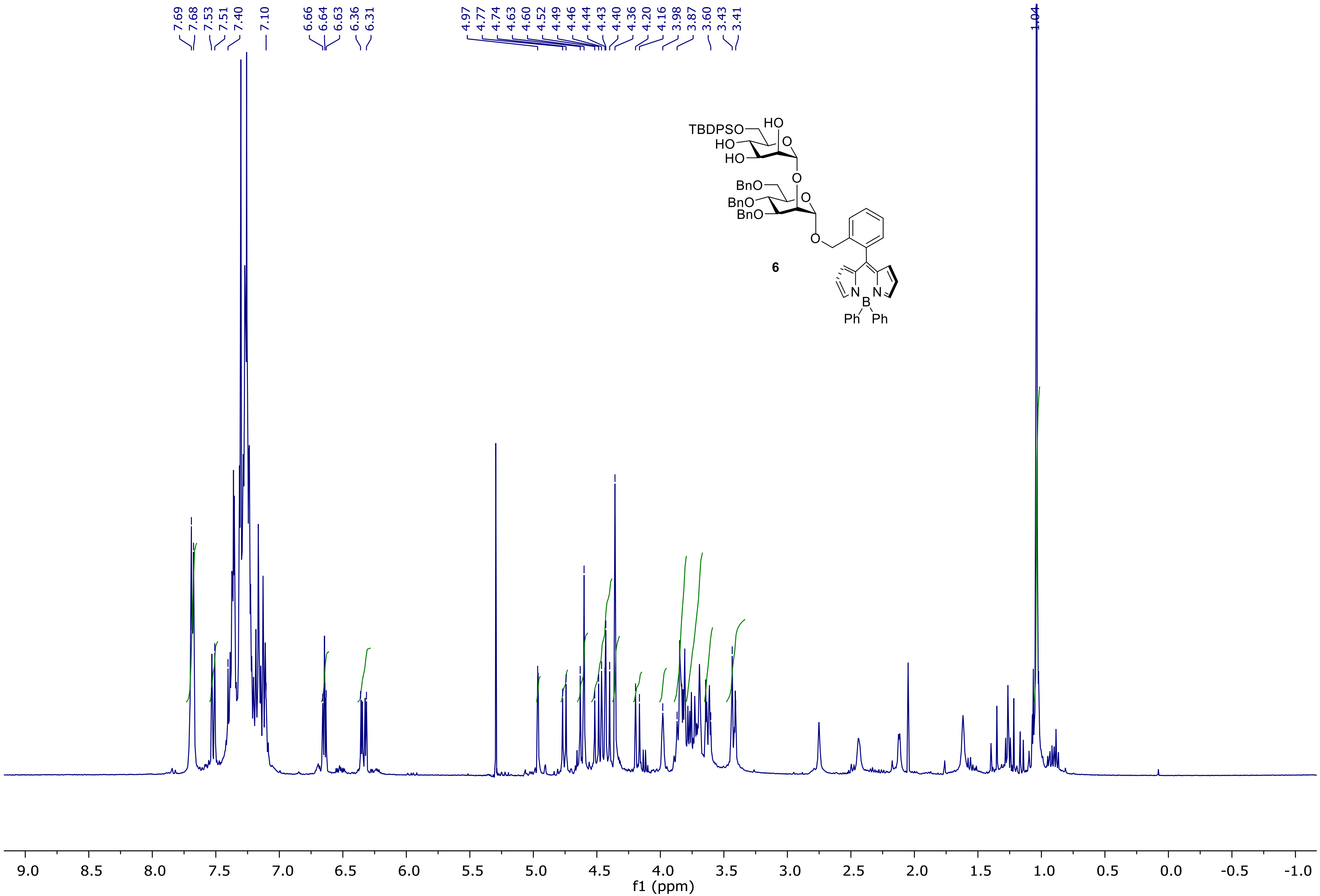

1H NMR (400 MHz, CDCl3)

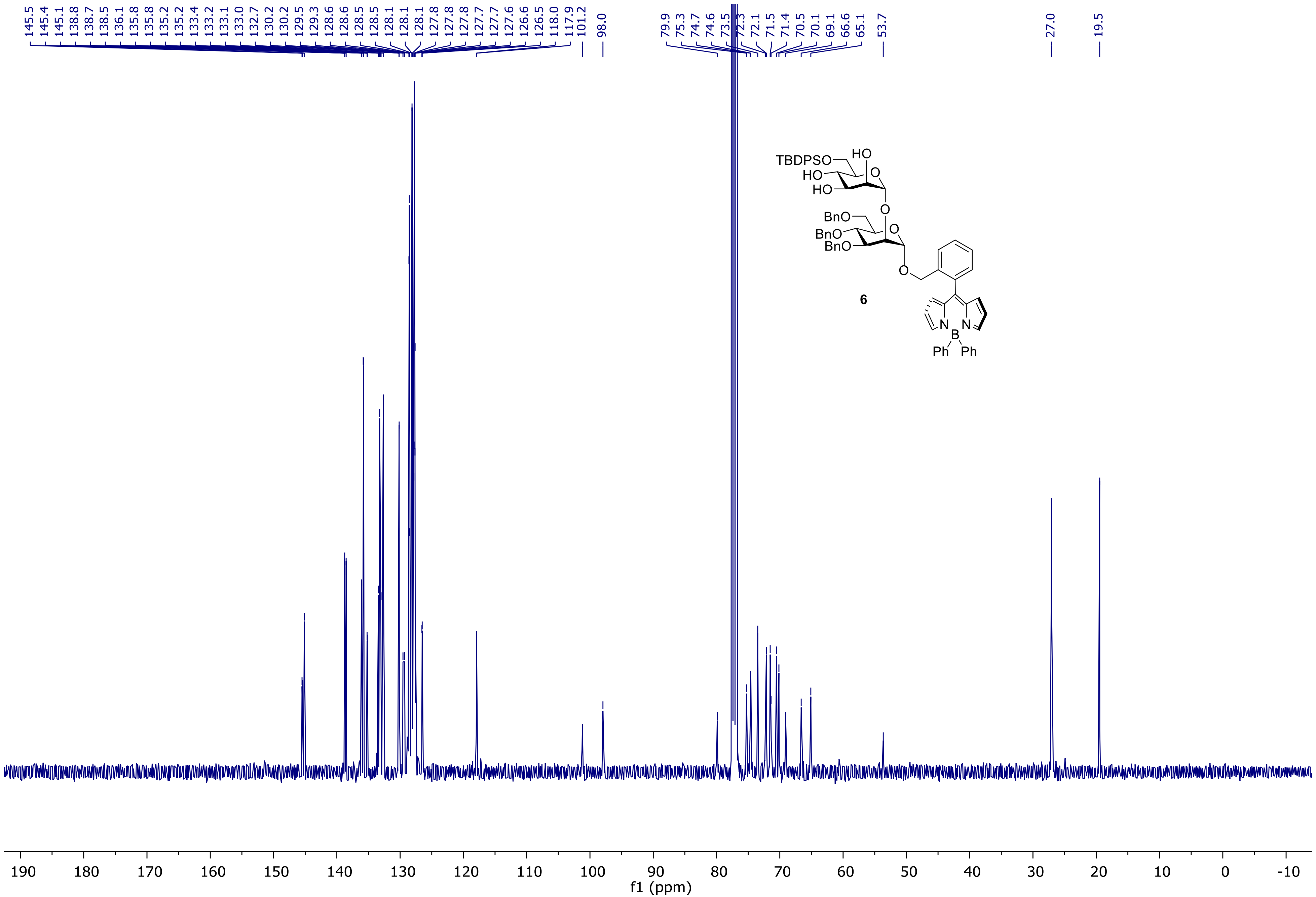

13C NMR (100 MHz, CDCl3)

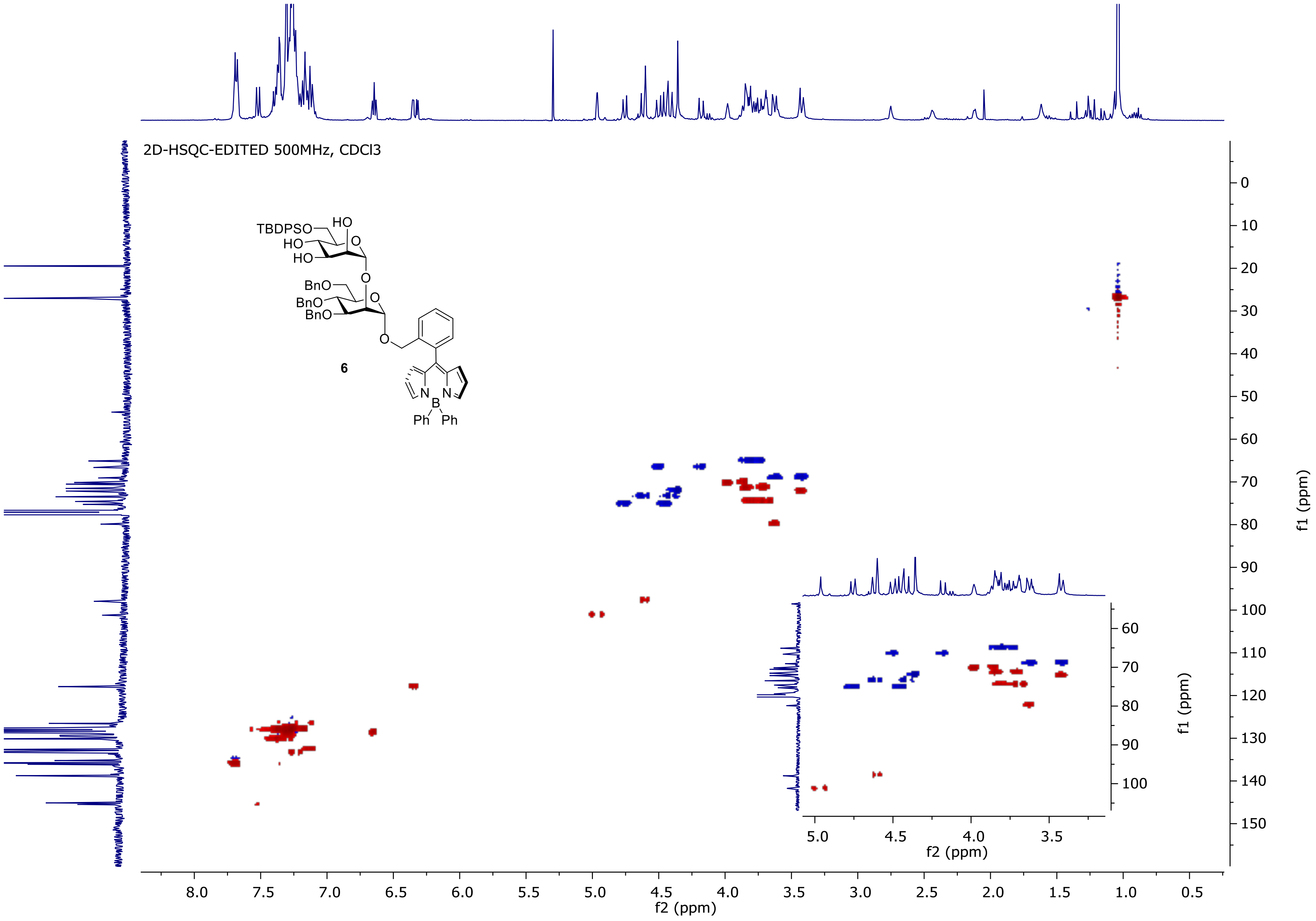
2D-HSQC-EDITED 500MHz, CDCl3
TBDPSO
HO
HO
HO
O
O
BnO
BnO
BnO
O
O
6
N
B
N
Ph
Ph
f1 (ppm)
f2 (ppm)

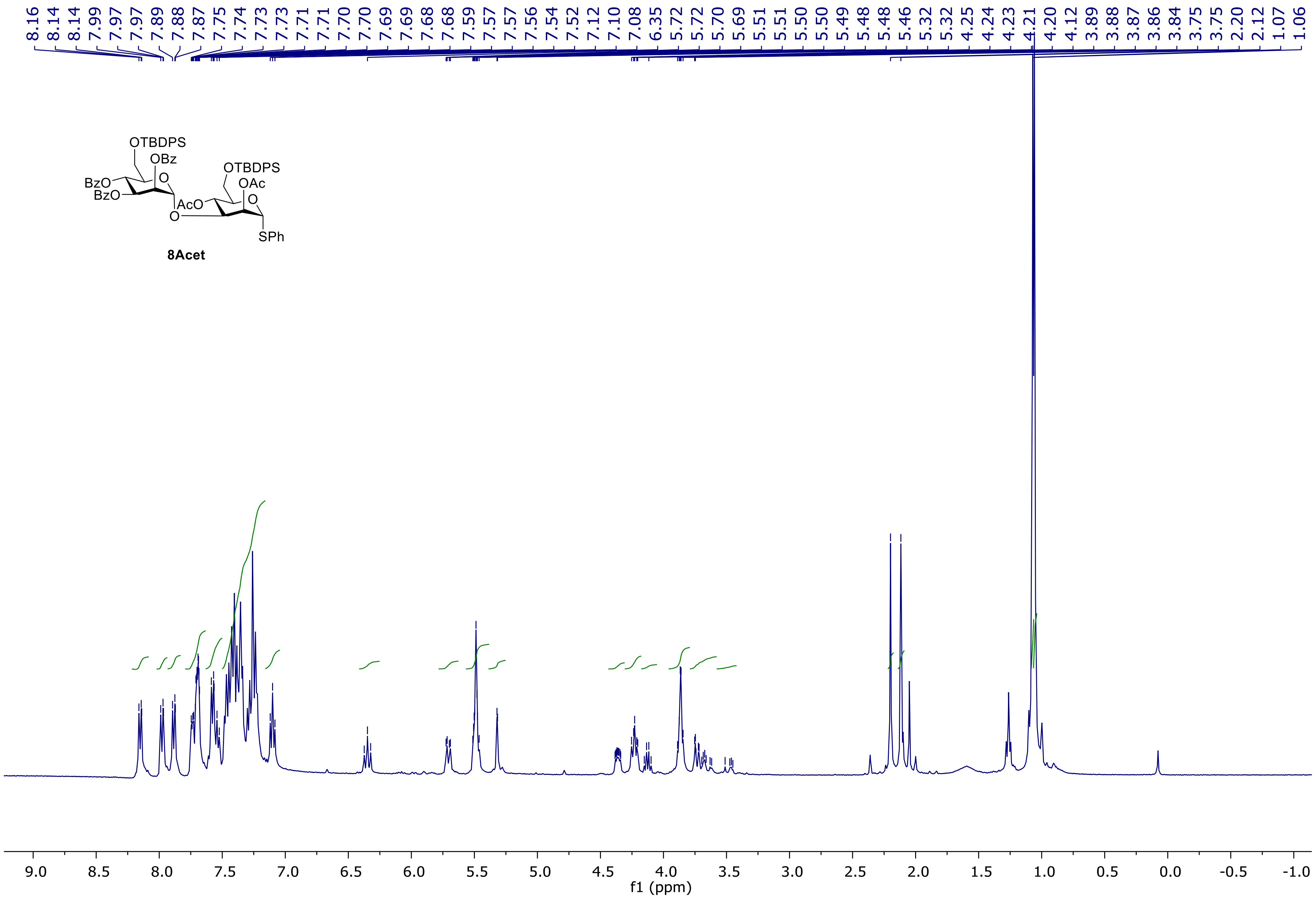

1H NMR (400 MHz, CDCl3)

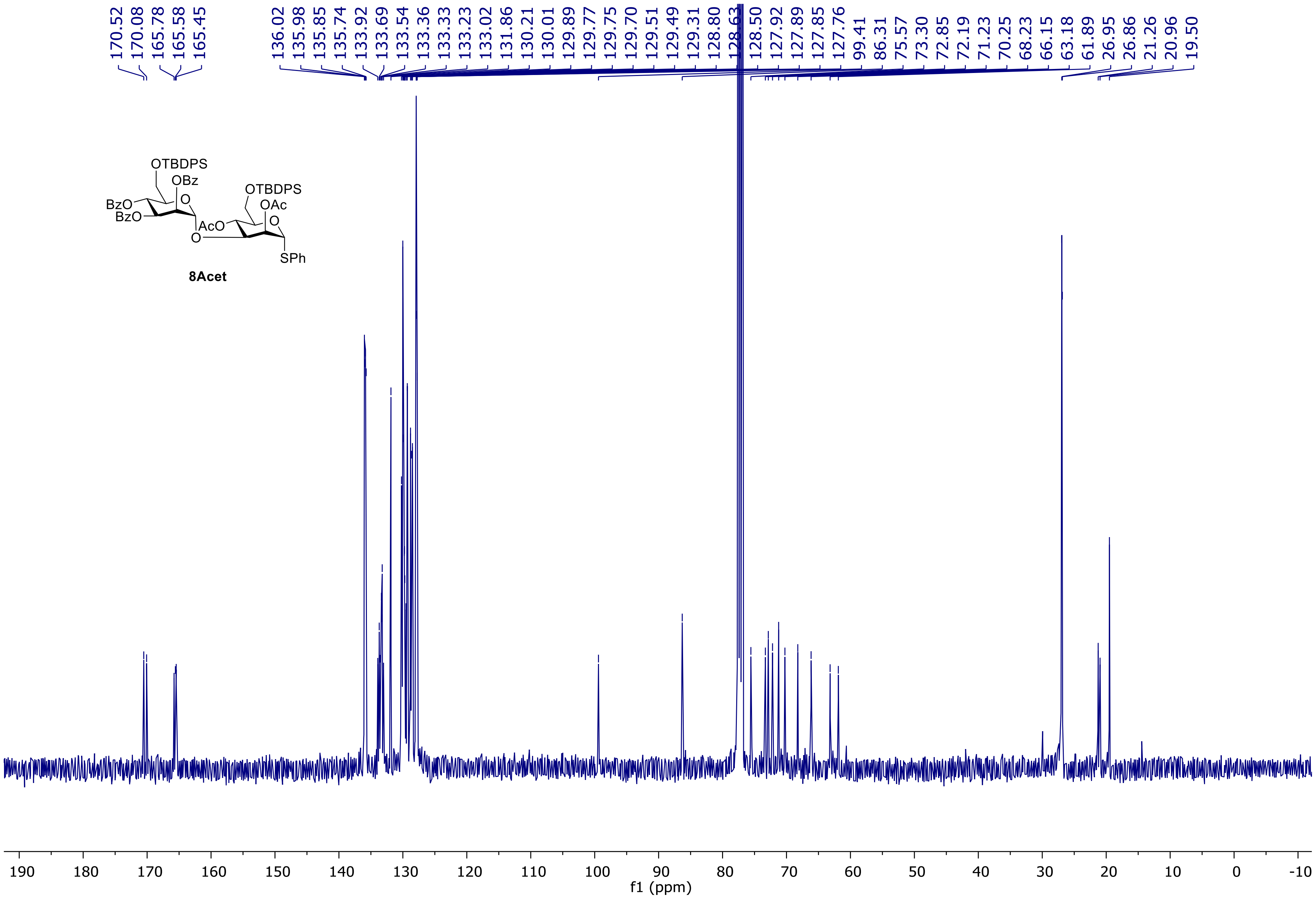

13C NMR (100 MHz, CDCl3)

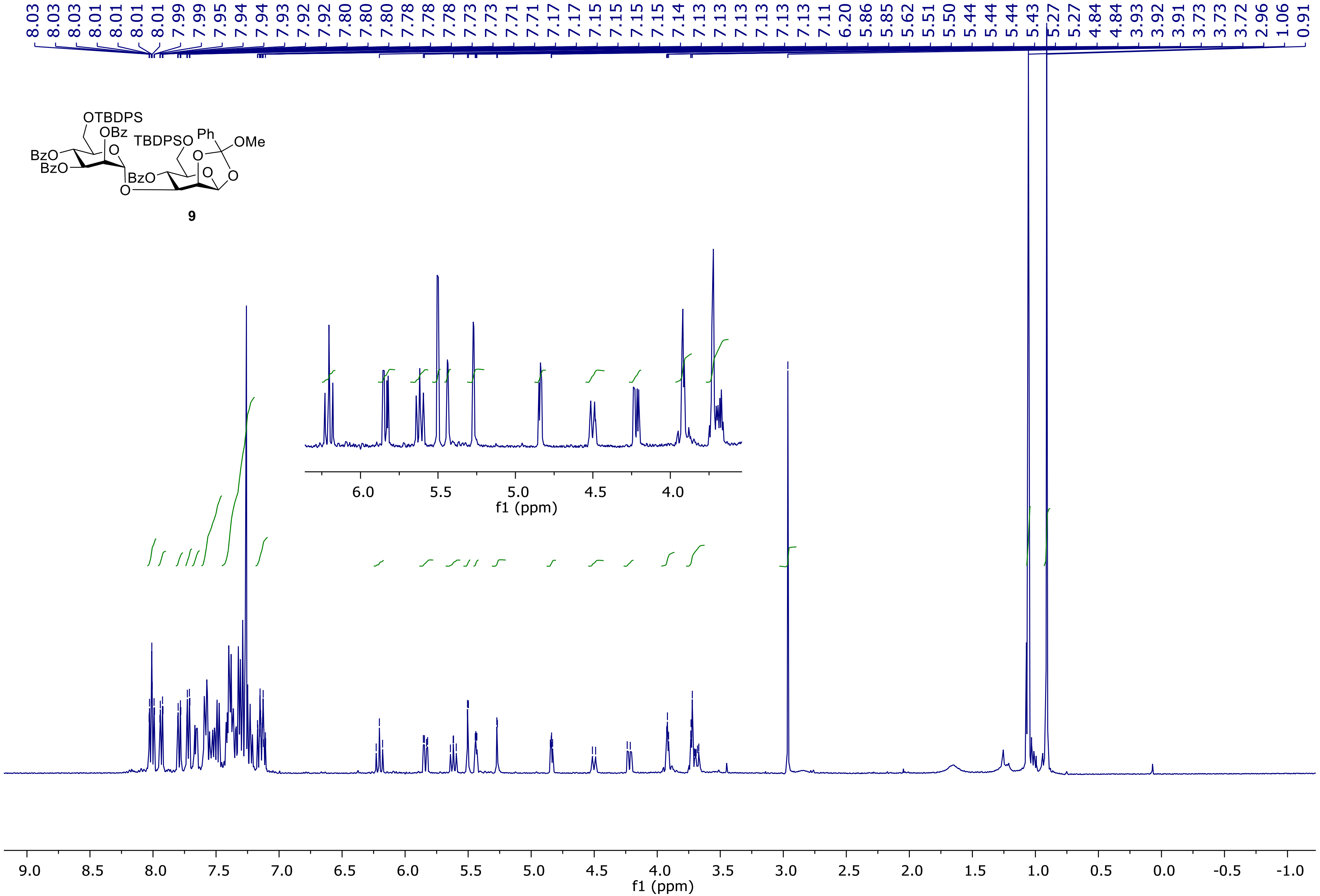

1H NMR (400 MHz, CDCl3)

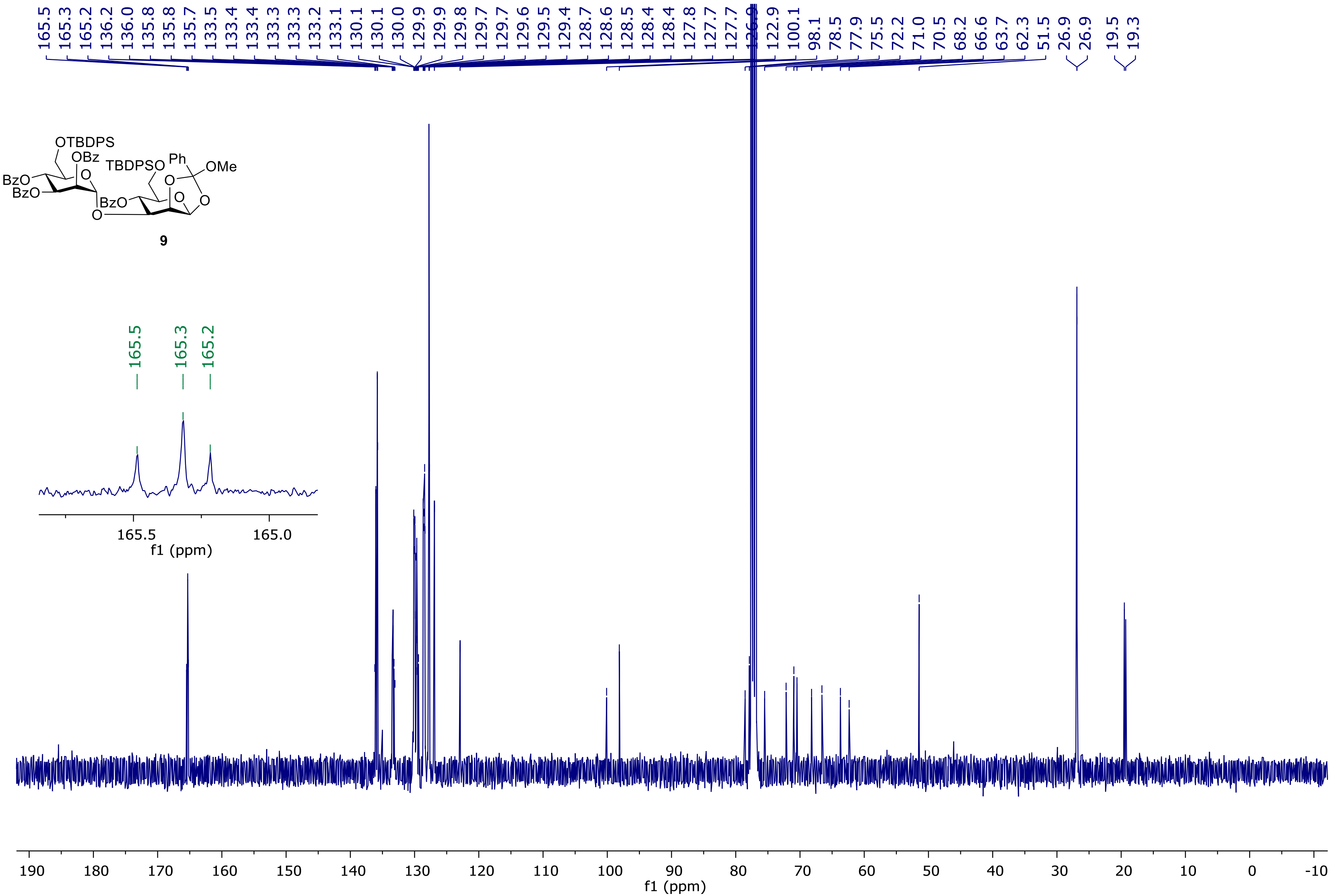

13C NMR (100 MHz, CDCl3)

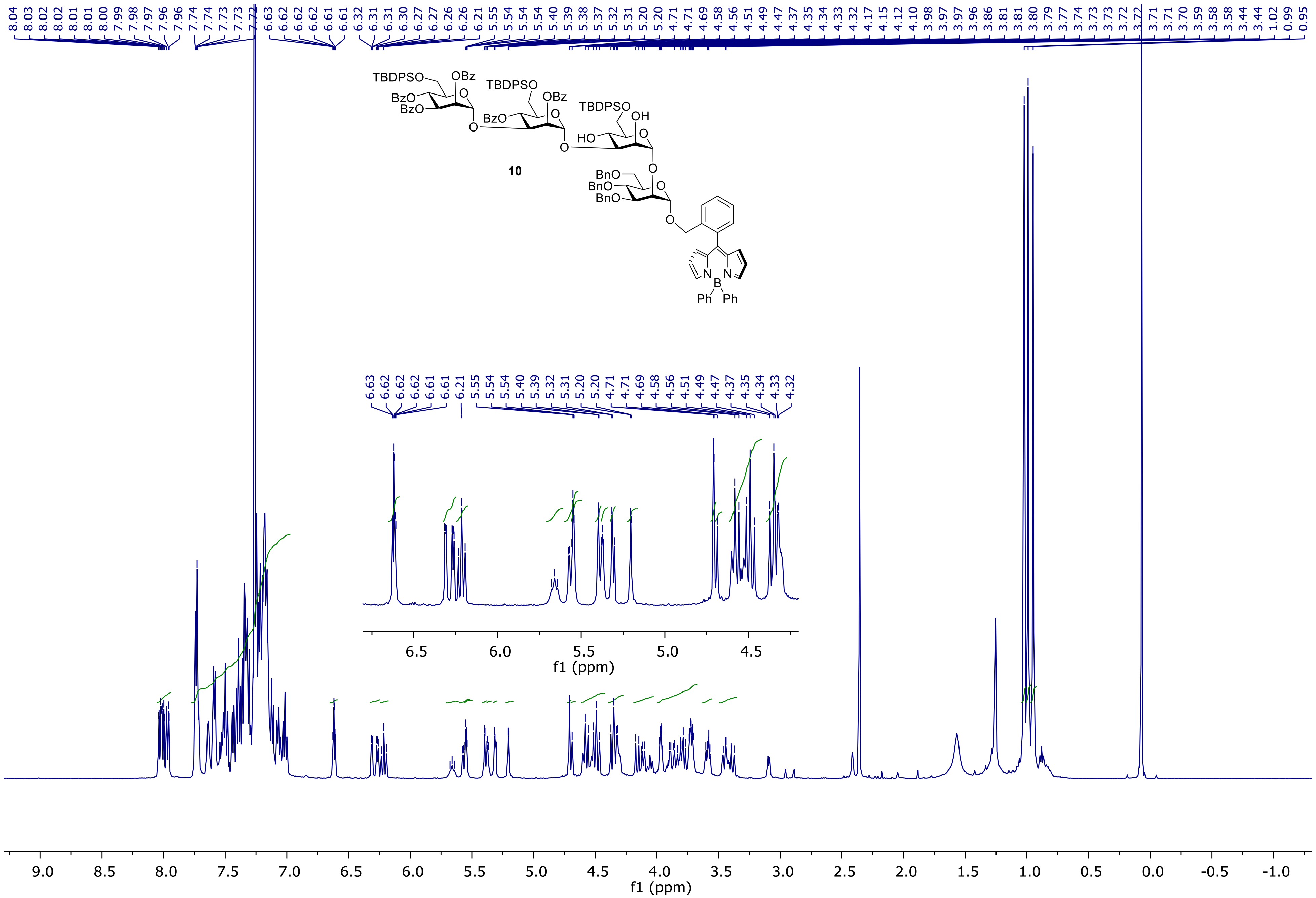

1H NMR (500 MHz, CDCl3)

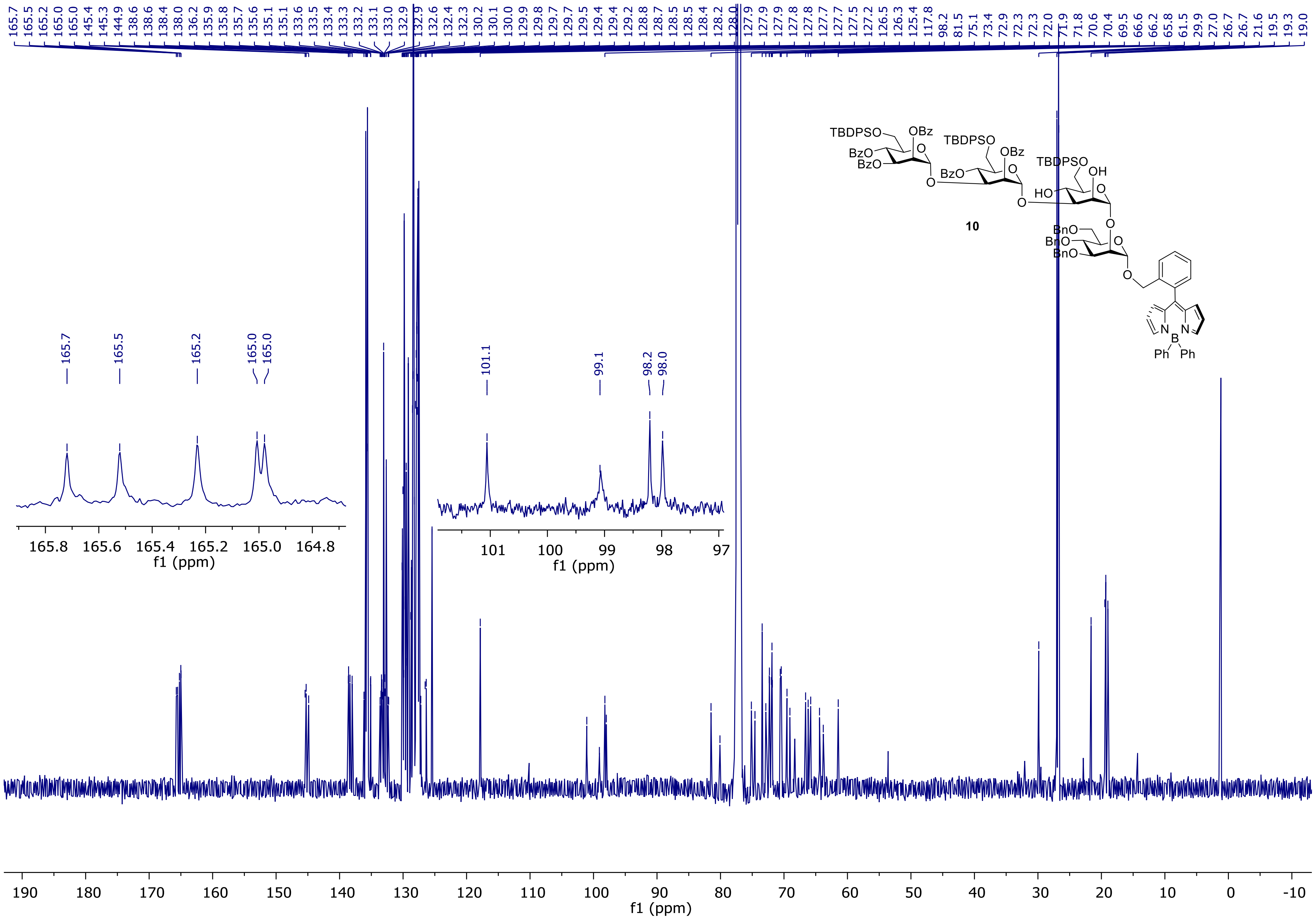
165.7
165.5
165.2
165.0
165.0
145.4
145.3
144.9
138.6
138.6
138.4
138.0
136.2
135.9
135.8
135.7
135.6
135.1
135.1
133.6
133.5
133.4
133.3
133.2
133.1
133.0
132.9
132.9
132.6
132.4
132.3
130.2
130.1
130.0
129.9
129.8
129.7
129.7
129.5
129.4
129.2
128.8
128.7
128.5
128.5
128.4
128.2
128.0
127.9
127.9
127.8
127.8
127.7
127.7
127.5
127.2
126.5
126.3
125.4
117.8
98.2
81.5
75.1
73.4
72.9
72.3
72.3
72.0
71.9
71.8
70.6
70.4
69.5
66.6
66.2
65.8
61.5
29.9
27.0
26.7
26.7
21.6
19.5
19.3
19.0
TBDPSO
OBz
BzO
BzO
TBDPSO
OBz
BzO
TBDPSO
OH
HO
10
BnO
BnO
BnO
N
B
N
Ph
Ph
165.8
165.6
165.4
165.2
165.0
164.8
f1 (ppm)
101.1
99.1
98.2
98.0
101
100
99
98
97
f1 (ppm)
190
180
170
160
150
140
130
120
110
100
90
80
70
60
50
40
30
20
10
0
-10
f1 (ppm)

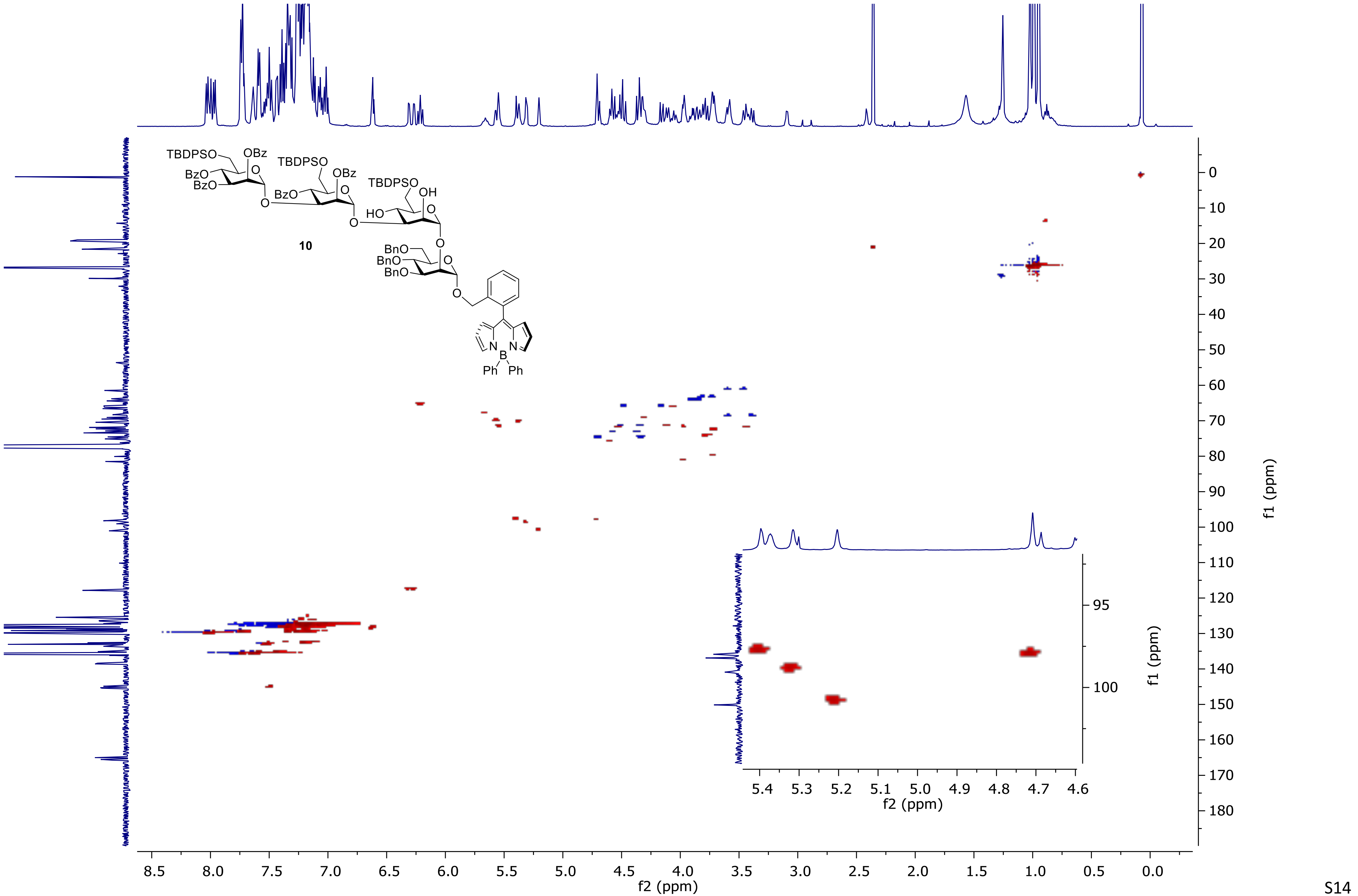

TBDPSO
OBz
BzO
BzO
TBDPSO
OBz
BzO
TBDPSO
OH
HO
10
BnO
BnO
BnO
N
B
N
Ph
Ph
f1 (ppm)
f2 (ppm)

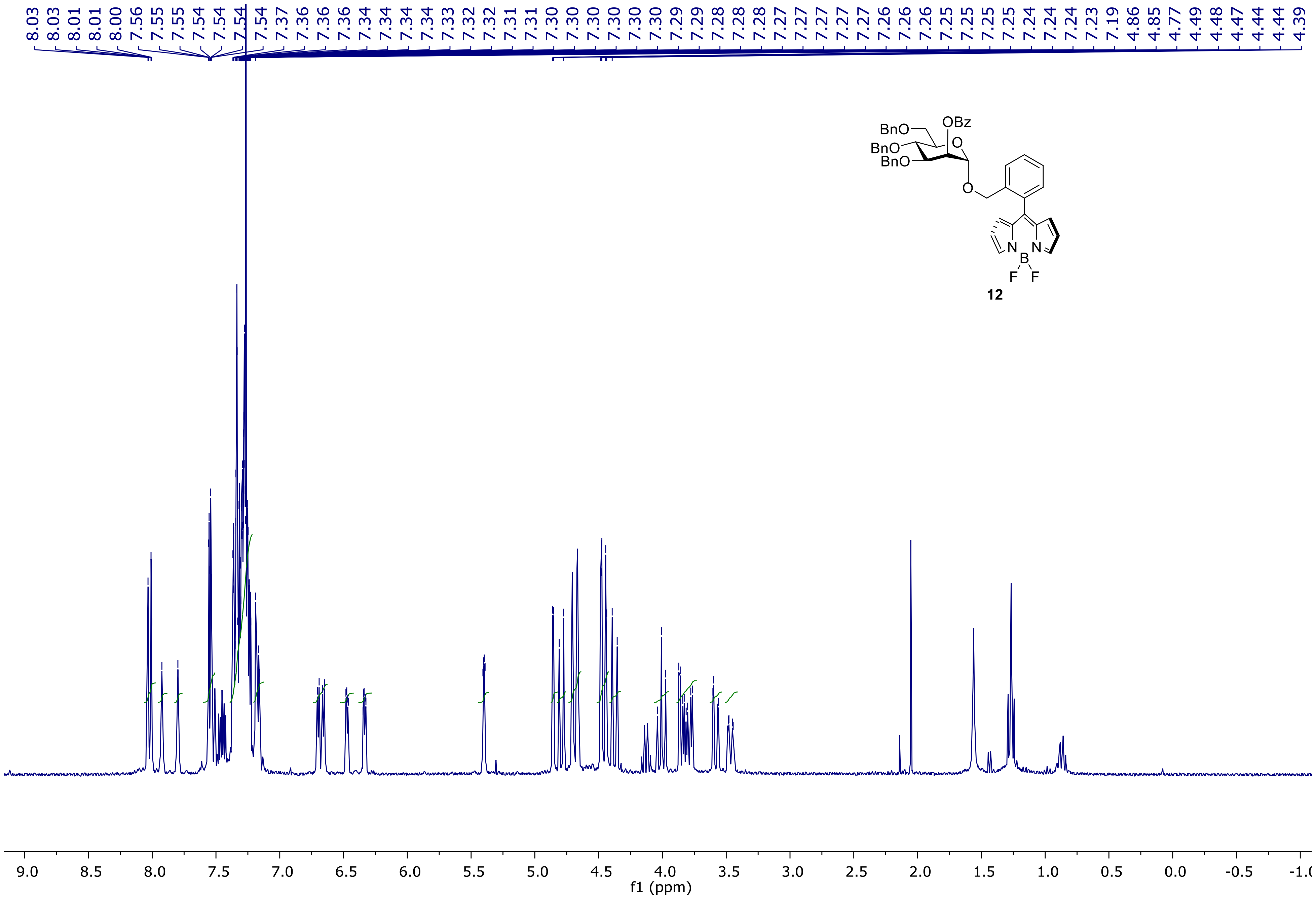

1H NMR (500 MHz, CDCl3)

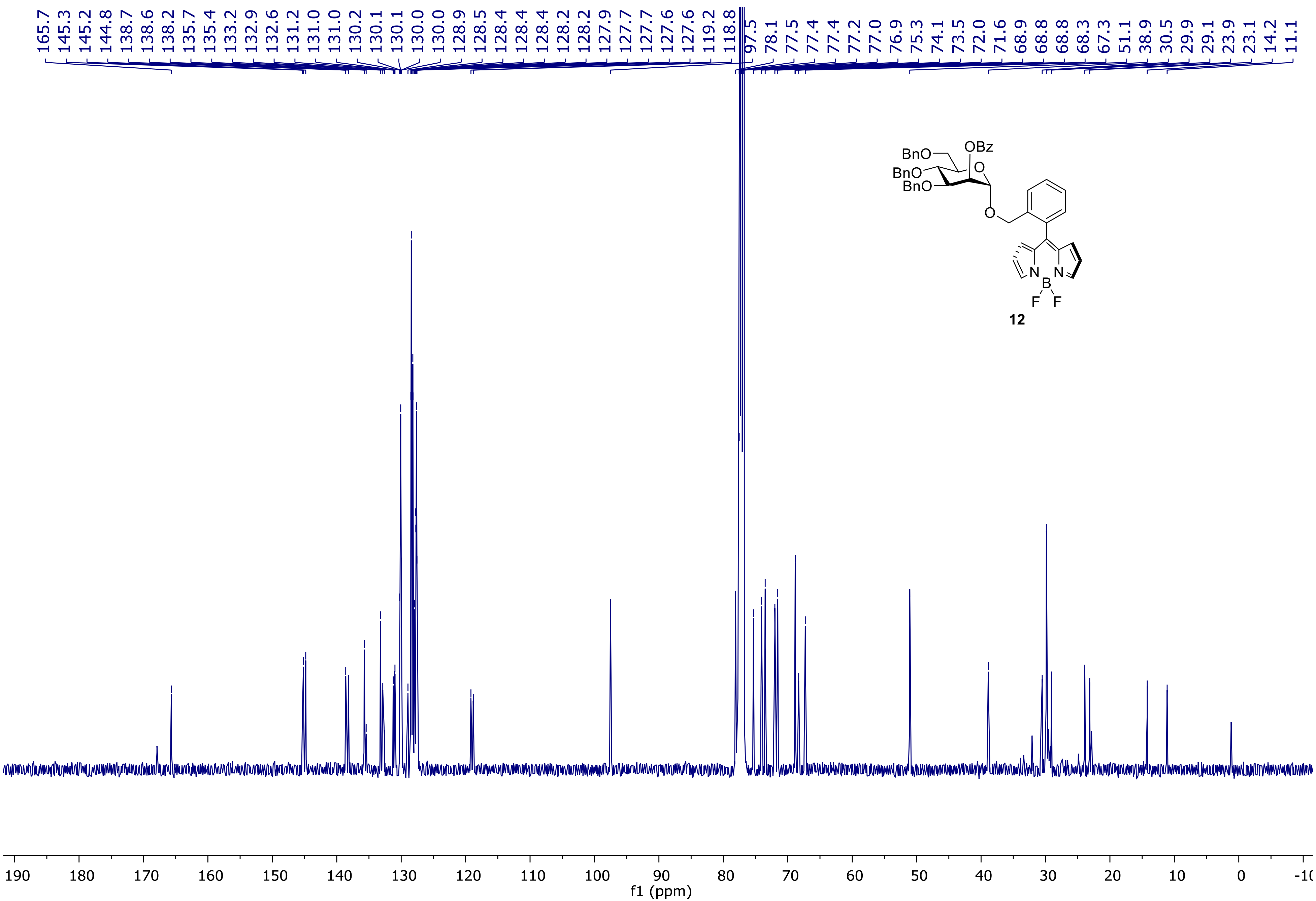

13C NMR (125 MHz, CDCl3)

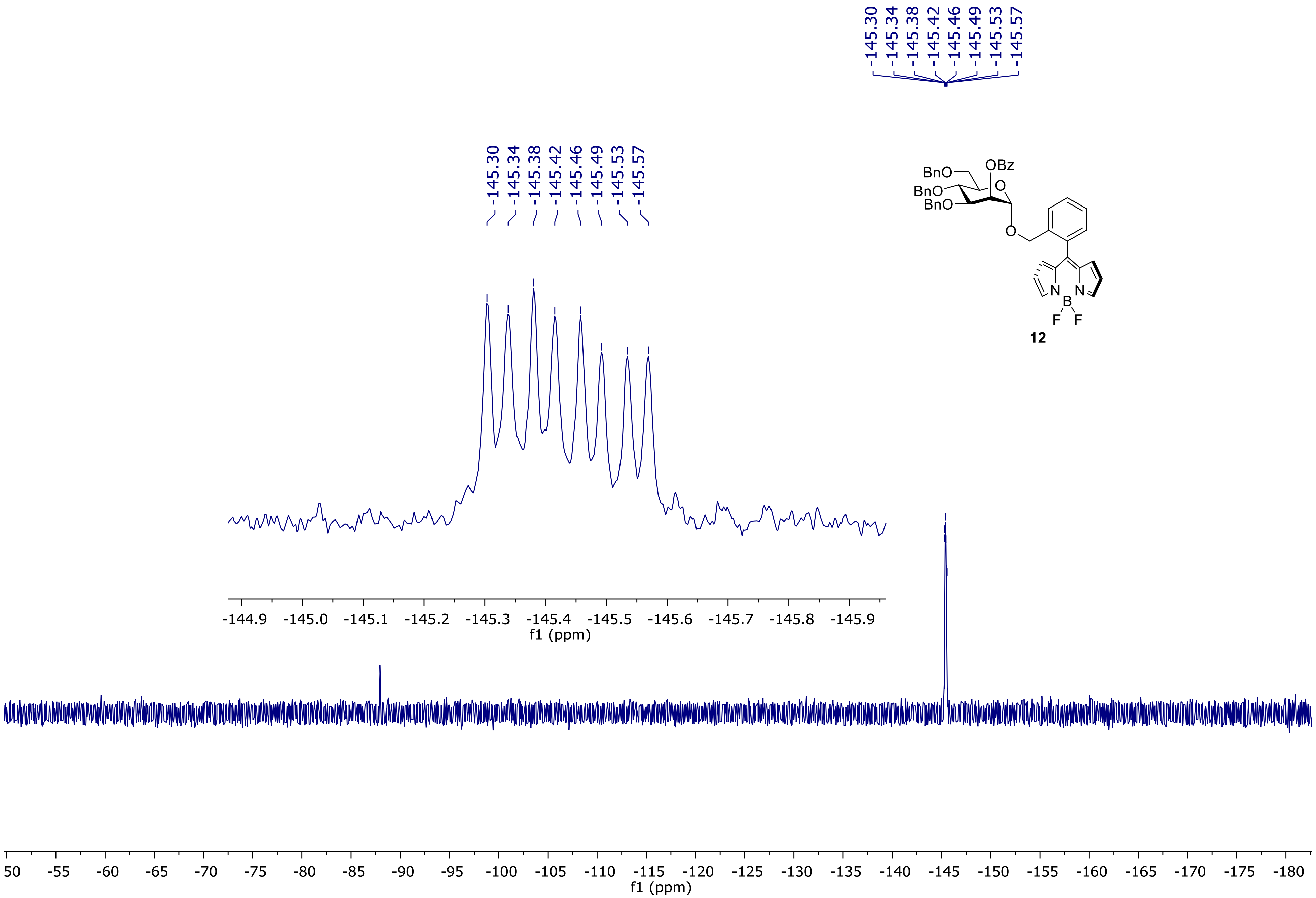

19F NMR (376 MHz, CDCl3)

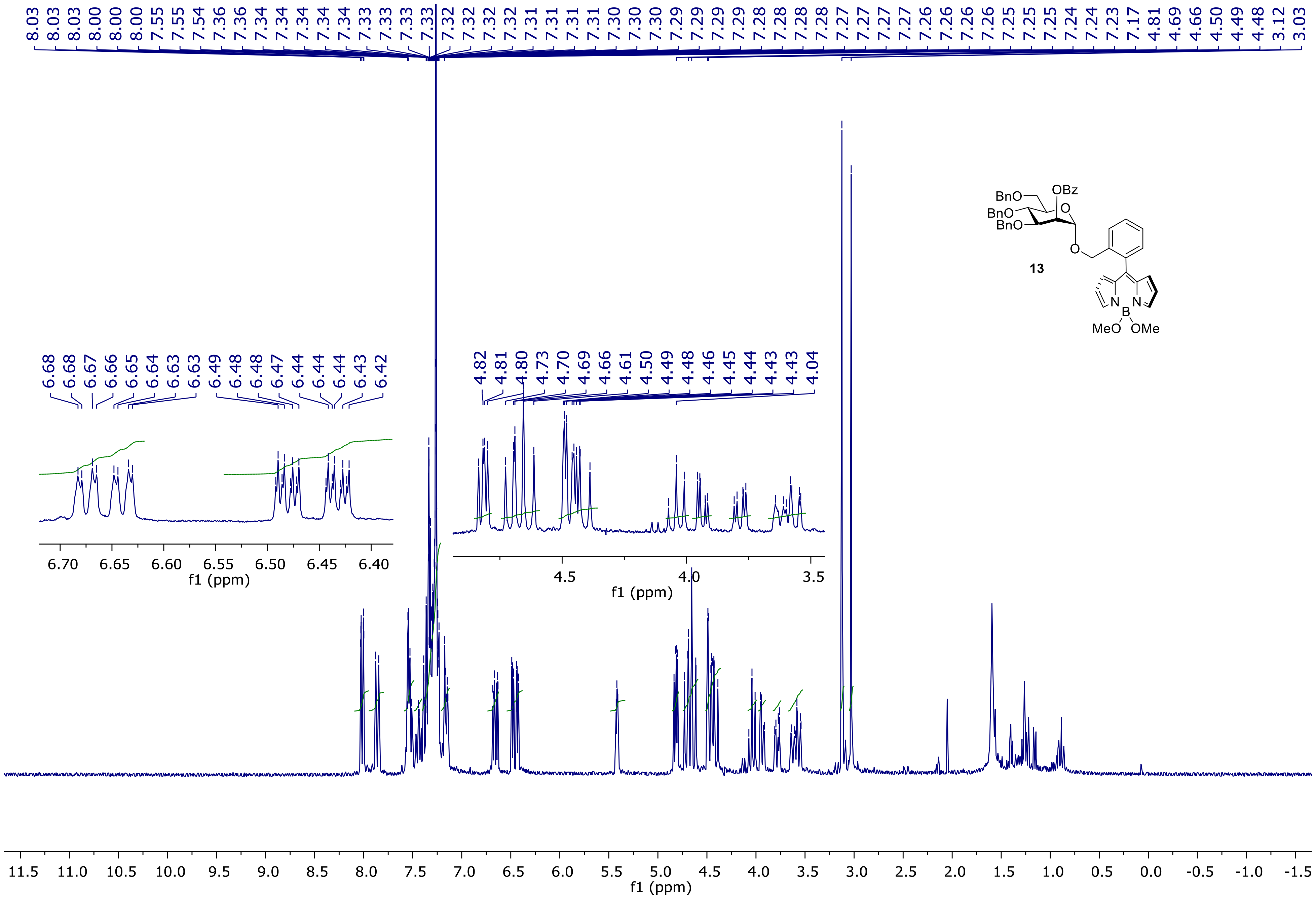

1H NMR (300 MHz, CDCl3)

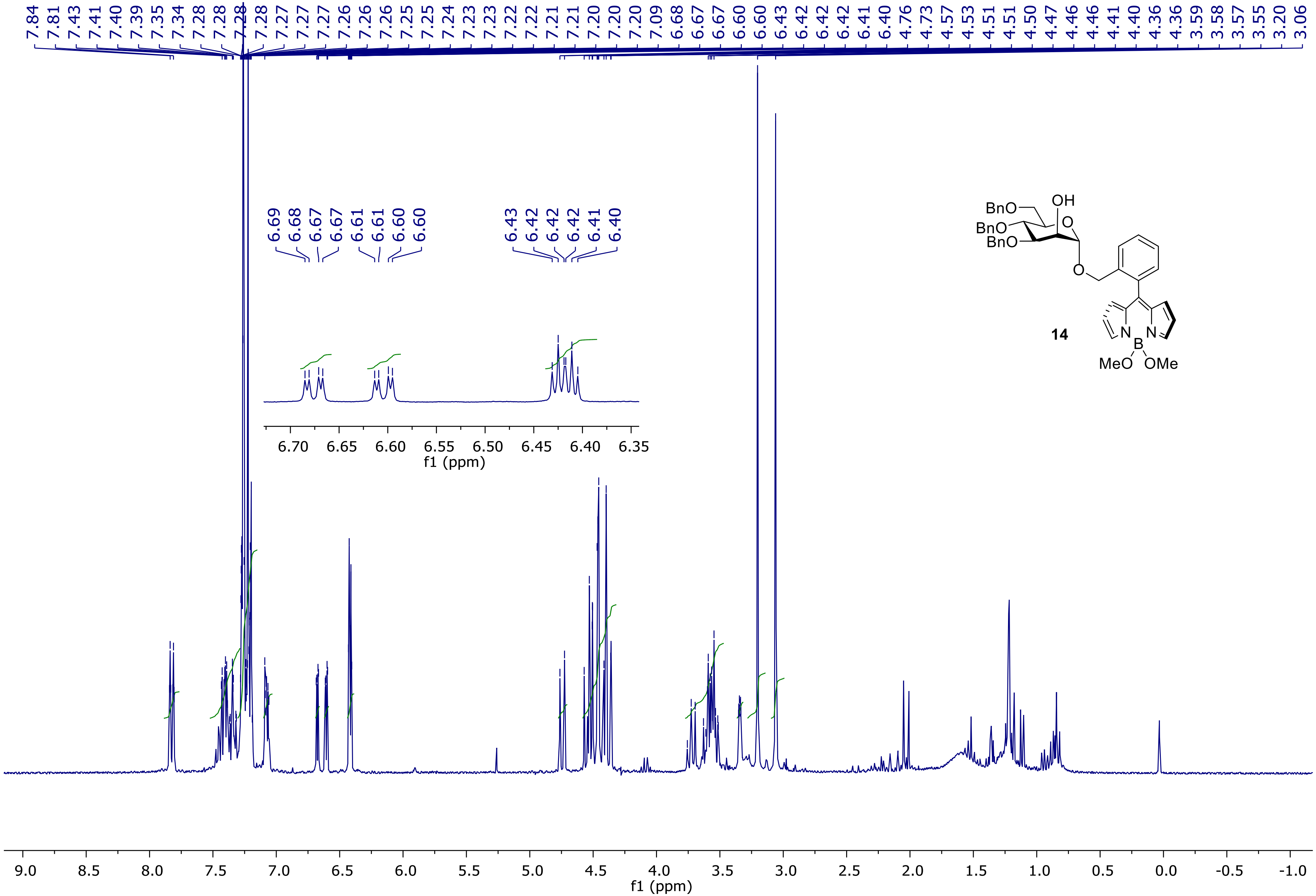

1H NMR (300 MHz, CDCl3)

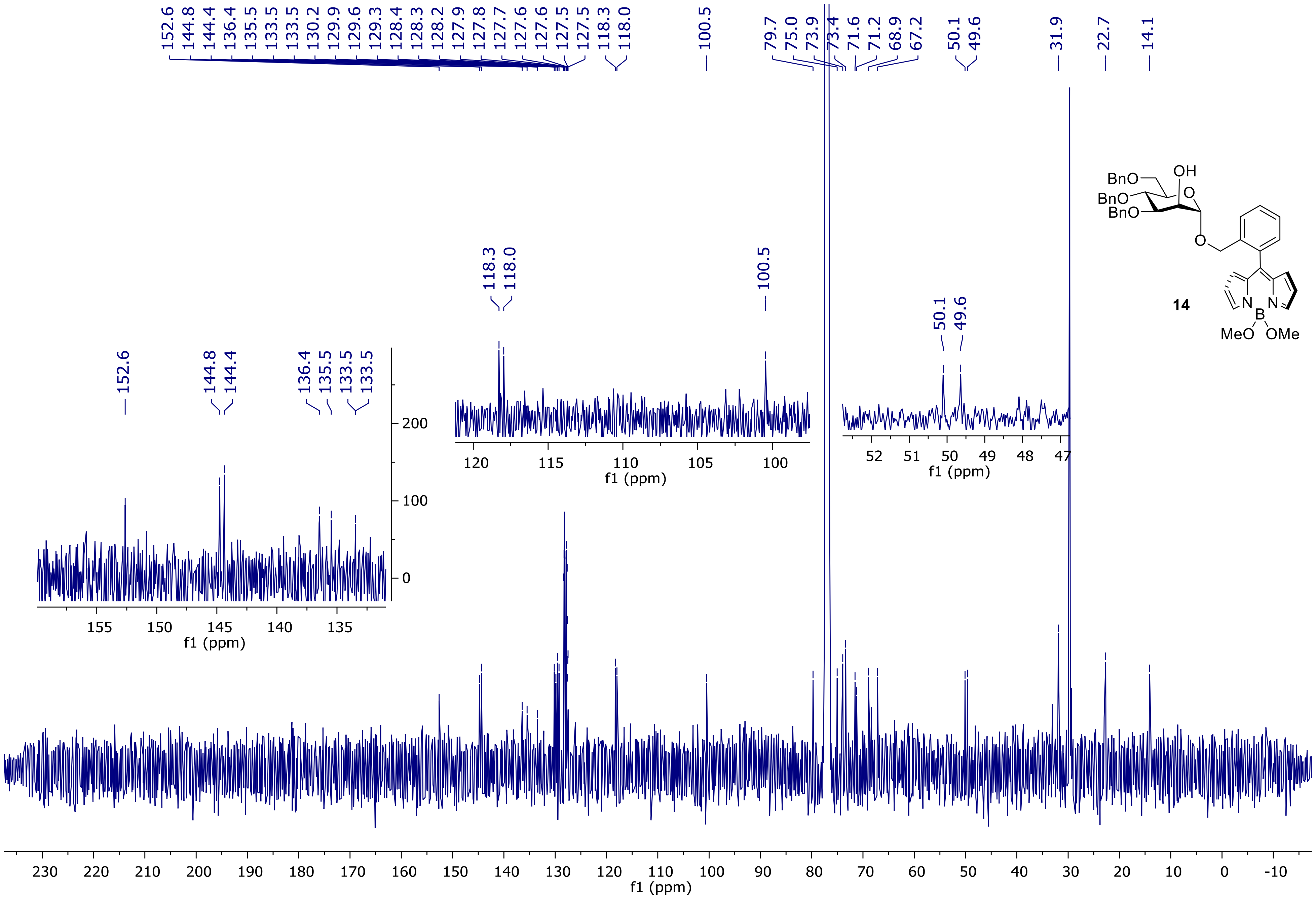

13C NMR (125 MHz, CDCl3)

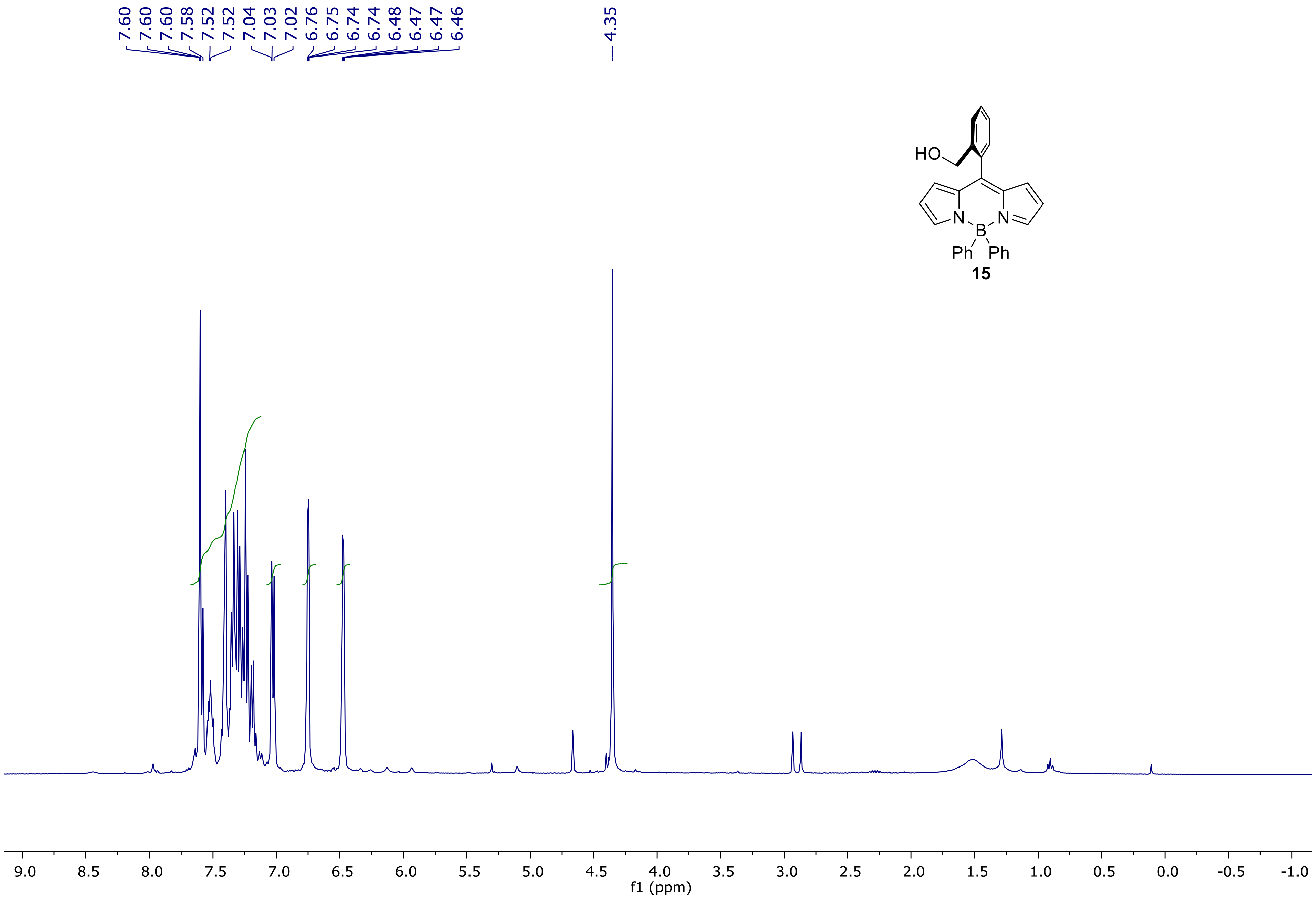

1H NMR (400 MHz, CDCl3)

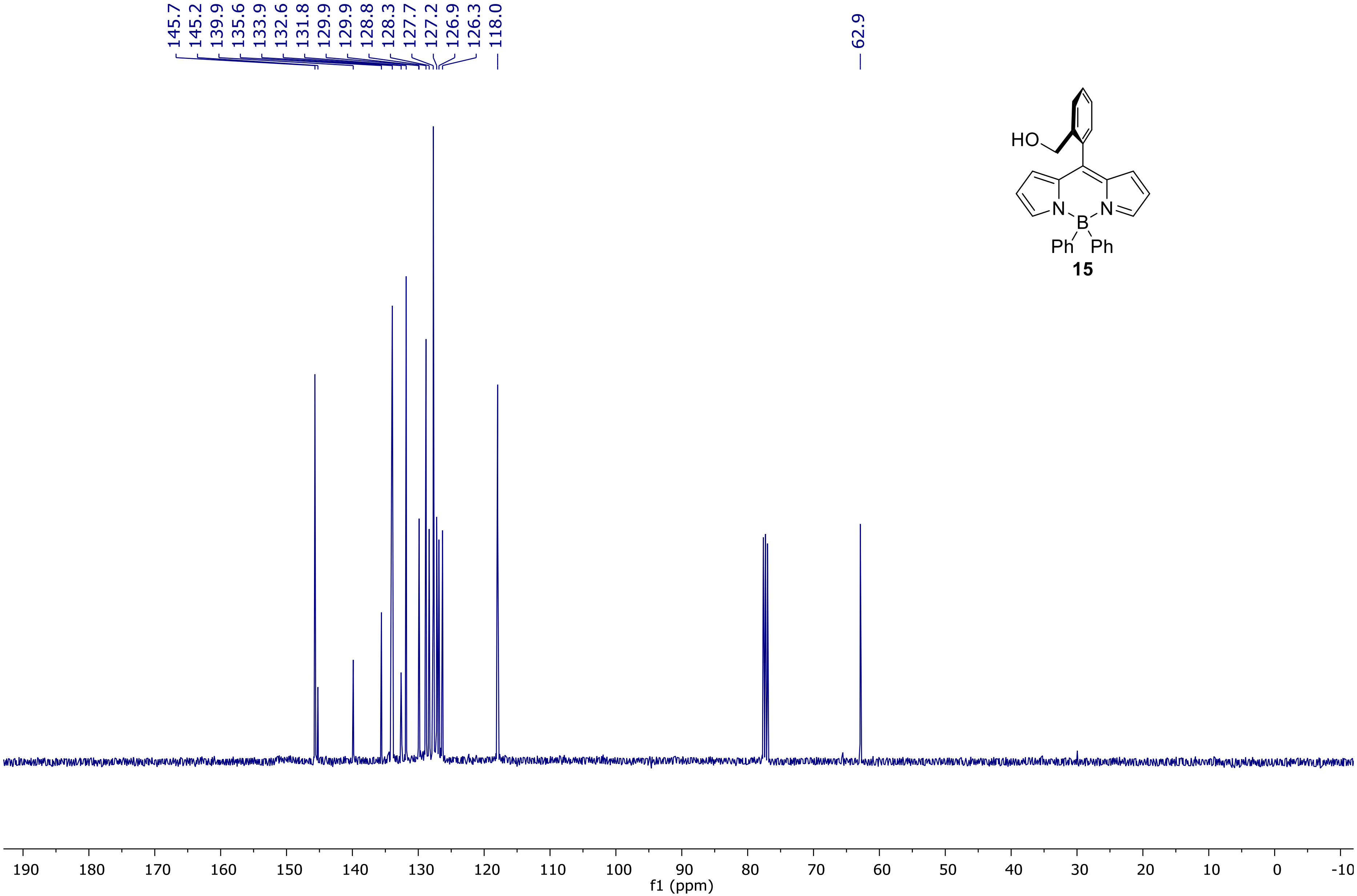

15

13C NMR (100 MHz, CDCl3)

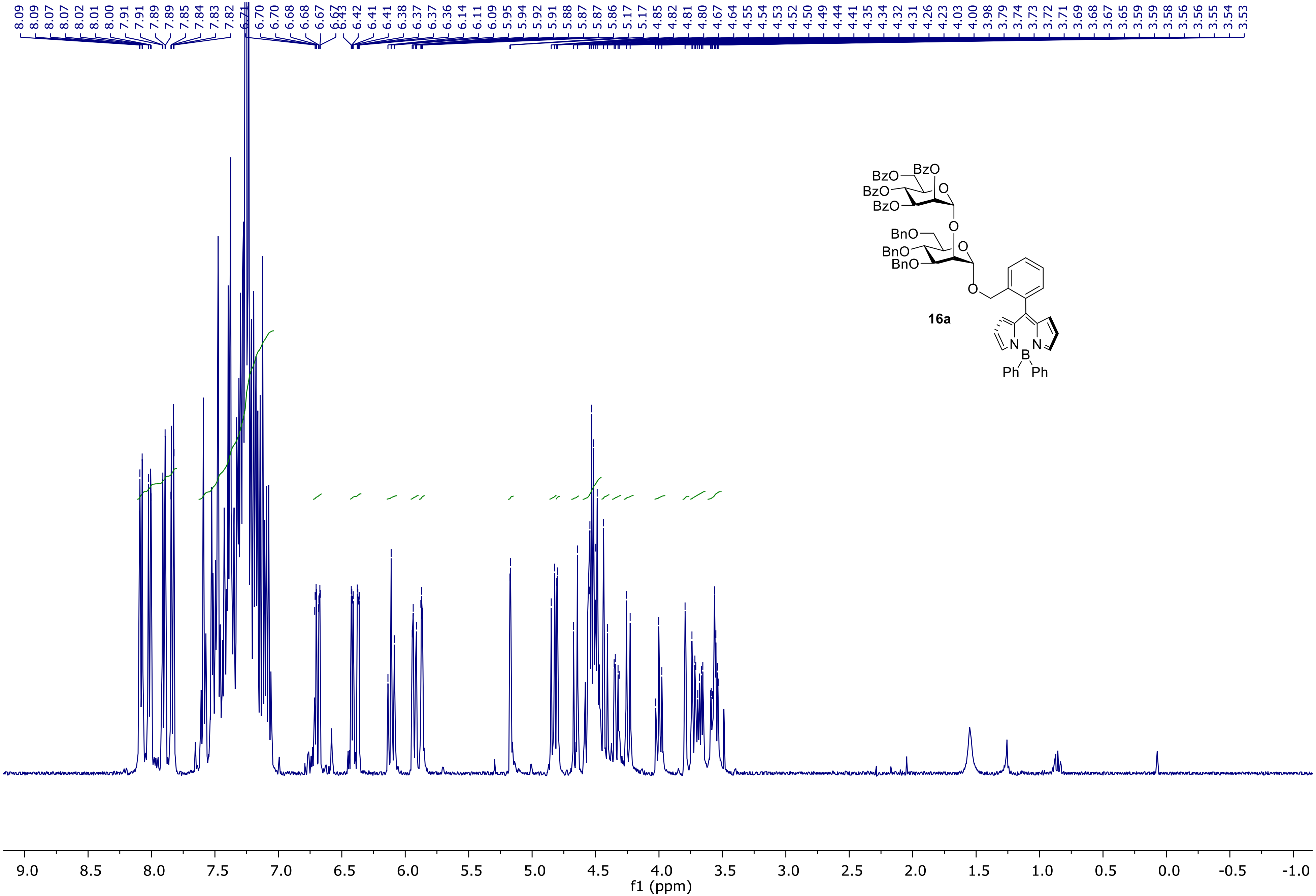

1H NMR (400 MHz, CDCl3)

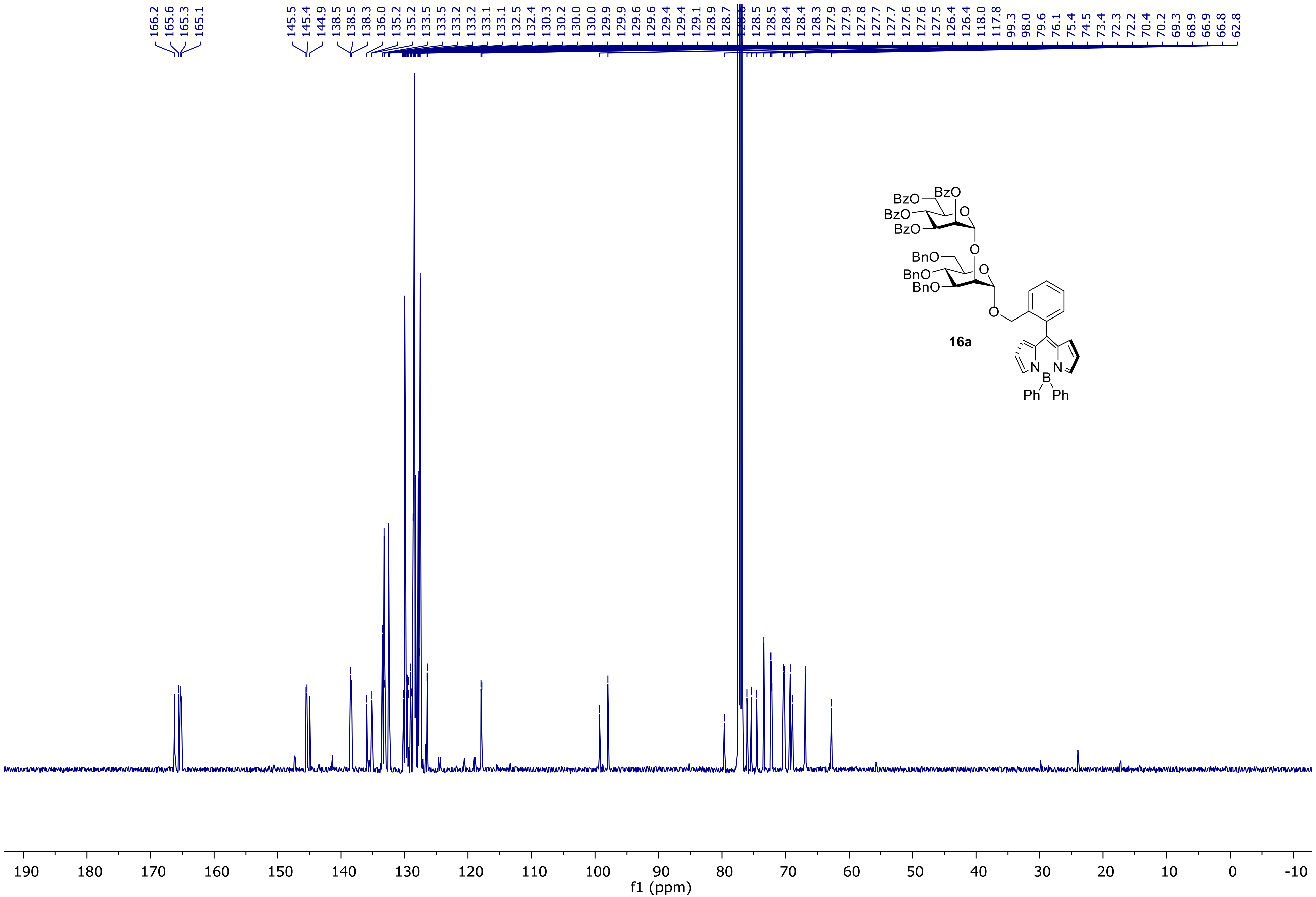

13C NMR (125 MHz, CDCl3)

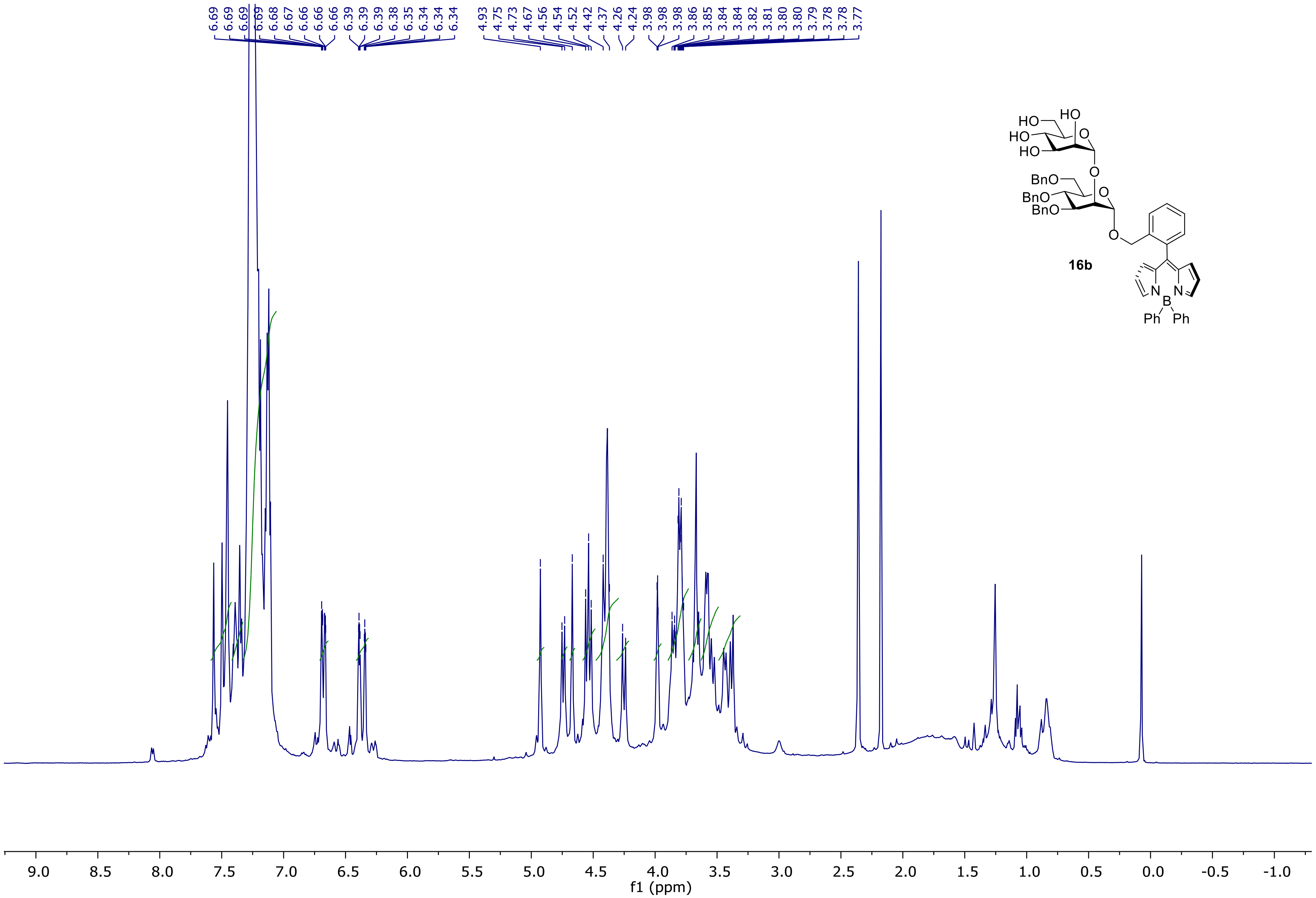

1H NMR (500 MHz, CDCl3)

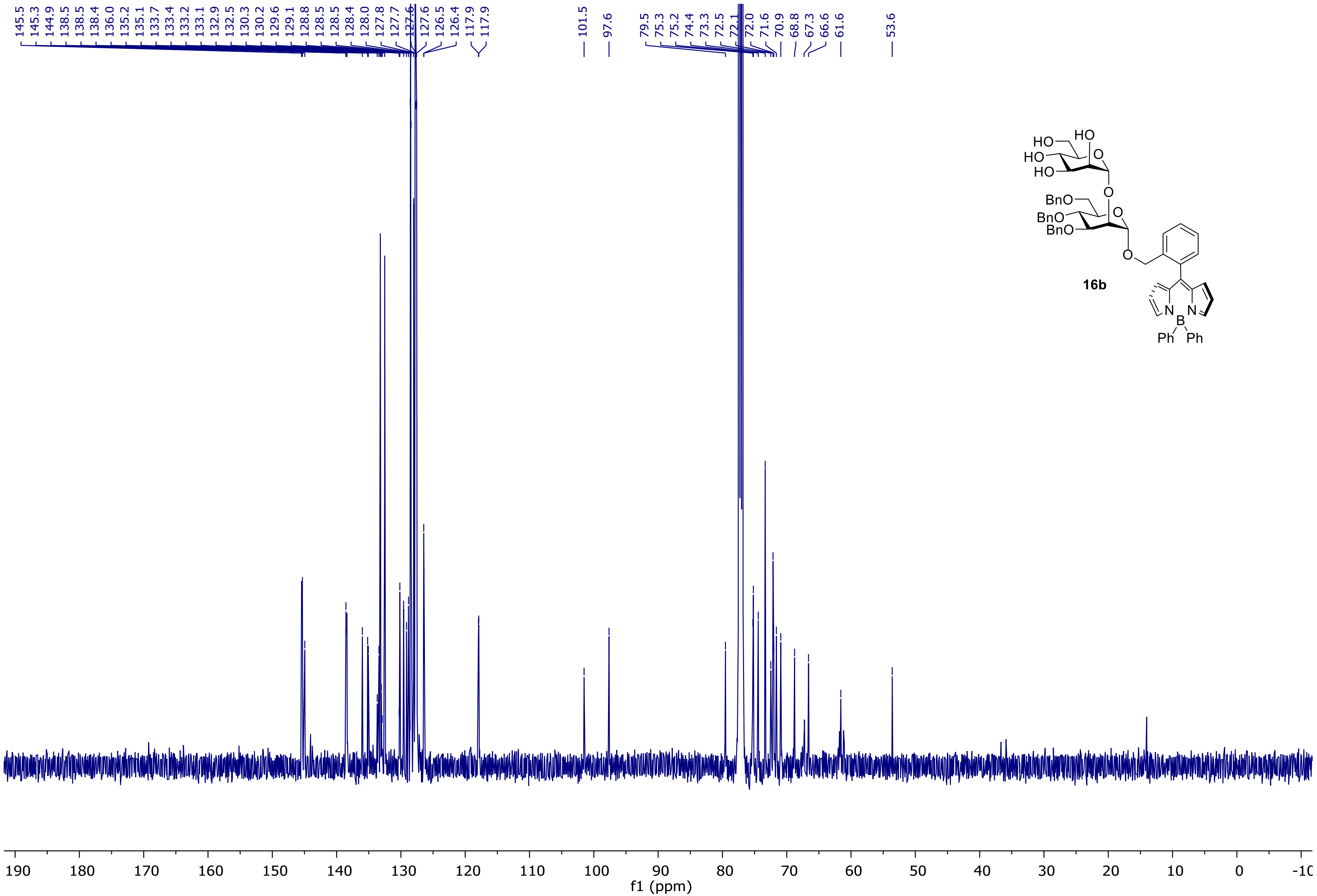

13C NMR (125 MHz, CDCl3)

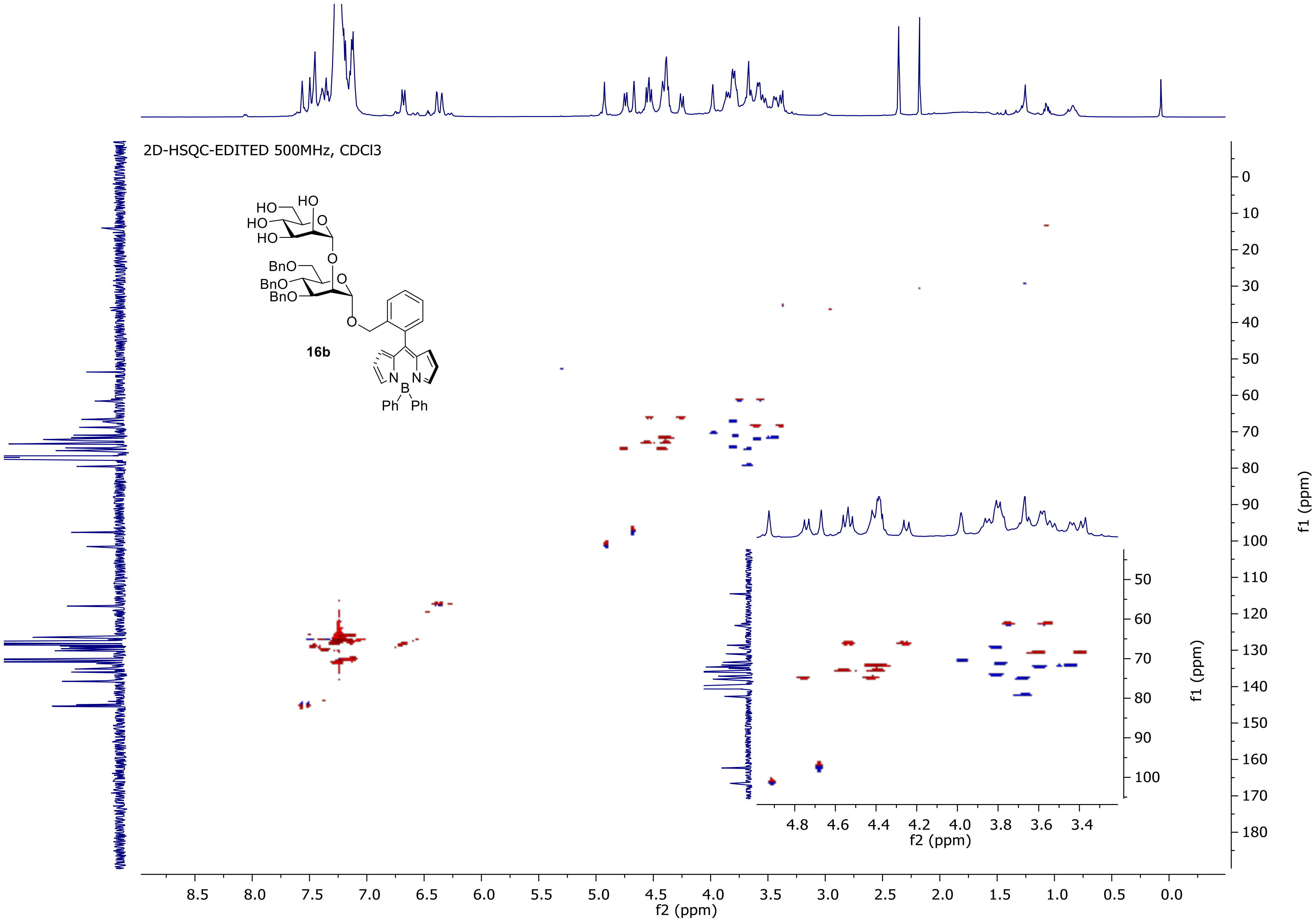
2D-HSQC-EDITED 500MHz, CDCl3
HO
HO
HO
HO
O
O
BnO
BnO
BnO
O
O
16b
N
B
N
Ph
Ph
f2 (ppm)
f1 (ppm)
f2 (ppm)
f1 (ppm)

Experimental: Photophysical and quantum mechanical properties

The photophysical properties were registered in diluted solutions (around 2×10^{-6} M), prepared by adding the corresponding solvent to the residue from the adequate amount of a concentrated stock solution in acetone, after vacuum evaporation of this solvent. UV-Vis absorption and fluorescence spectra were recorded on a Varian model CARY 4E spectrophotometer and an Edinburgh Instruments spectrofluorimeter (model FLSP 920), respectively. Fluorescence quantum yields (ϕ) were obtained using PM546 (laser grade from Exciton, ϕ = 0.85 in ethanol) as reference, from corrected spectra (detector sensibility to the wavelength). The values were corrected by the refractive index of the solvent. Radiative decay curves were registered with the time correlated single-photon counting technique as implemented in the aforementioned spectrofluorimeter. Fluorescence emission was monitored at the maximum emission wavelength after excitation by means of a Fianium pulsed laser (time resolution of picoseconds) with tunable wavelength. The fluorescence lifetime (τ) was obtained after the deconvolution of the instrumental response signal from the recorded decay curves by means of an iterative method. The goodness of the exponential fit was controlled by statistical parameters (chi-square, Durbin-Watson and the analysis of the residuals). For the fluorescent lifetime with a biexponential character, the amplitude average lifetime was calculated as follows; $<\tau> = \Sigma A_i\tau_i$, where A_i is the preexponential factor, which accounts for the contribution of each exponential to the whole decay. The radiative (k_{fl}) and non-radiative (k_{nr}) rate constants were calculated from the fluorescence quantum yield and average lifetime; $k_{fl} = \phi/<\tau>$ and $k_{nr} = (1-\phi)/<\tau>$.

Ground state geometries were optimized with the wb97xd range-separated hybrid functional, within the Density Functional Theory (DFT), using the triple valence basis set with two polarization functions (6-311g**). First excited state geometry was optimized by means of the Time Dependent method (TD-DFT) at the same calculation level. The geometries were considered as energy minimum when the corresponding frequency analysis did not give any negative value. Such geometry minimizations were carried out considering the solvent effect (ethyl acetate) by means of the Polarizable Continuum Model (PCM). All the calculations were conducted under the Gaussian 16.

Laser experiments

Liquid solutions of dyes were contained in 1 cm optical-path rectangular quartz cells carefully sealed to avoid solvent evaporation during experiments. The liquid solutions were transversely pumped at 355 nm, with 5 mJ, 8 ns FWHM pulses from the third-harmonic of Q-switched Nd:YAG laser (Spectron SL282G), at a repetition rate of up to 10 Hz. The exciting pulses were line-focused onto the cell, providing pump fluences on the active medium in the range 110 - 180 mJ/cm^2. The oscillation cavity (2 cm length) consisted of a 90% reflectivity aluminum mirror, with the lateral face of the cell as output coupler. The energy of dye and pump laser pulses was measured with GenTec ED-100A and ED-200 pyroelectric energy meters. The spectral characteristics of the laser emission were determined by collecting a fraction of the emission by an optical fiber attached to the input slit of a spectrograph/monochromator (SpectraPro-300i, Acton Research Corporation) equipped with a charge-coupled device (CCD) /SpectruMM:GS 128B). The photostability of the dyes in liquid solution was evaluated by transversally irradiating under lasing conditions 100 µL of a solution in ethyl acetate and monitoring the decrease in laser-induced intensity as a function of the number of pump pulses at a given repetition rate. The laser emission was monitored perpendicular to the exciting beam, collected by an optical fiber, and imaged onto the input slit of the monochromator and detected with the CCD. The laser emission was recorded by feeding the signal to the boxcar (Stanford Research, model 250) to be integrated before being digitized and processed by a computer. Each experience was repeated at least three times. The estimated error in the energy and photostability measurements was 10%.

Table S1. Photophysical properties of dye ***15*** *and its glycosylated derivatives* ***10*** *and* ***16a*** *(bearing a tetrasaccharide and a disaccharide respectively) in diluted solutions of different solvents*

	λ_{ab} (nm)	ε_{max} ($10^4 M^{-1} cm^{-1}$)	λ_{fl} (nm)	$\Delta\nu_{St}$ (cm^{-1})	ϕ	τ (ns)	$<\tau>$ (ns)	k_{fl} ($10^8 s^{-1}$)	k_{nr} ($10^8 s^{-1}$)
**15**									
c-hex	500.0	5.3	516.5	640	0.32	2.40(60%) 3.49(40%)	2.83	1.13	2.40
Et. Ac.	497.0	4.8	511.5	570	0.22	2.63(84%) 3.88(16%)	2.83	0.77	2.75
MeOH	496.5	4.8	513.0	650	0.21	2.48(90%) 5.12(10%)	2.74	0.76	2.88
**10**									
c-hex	502.5	4.2	518.0	595	0.30	3.21(87%) 5.88(13%)	3.60	0.83	1.94
Et. Ac.	500.5	4.1	515.0	565	0.31	3.09(83%) 5.89(17%)	3.57	0.88	1.92
MeOH	499.5	4.2	513.5	550	0.28	3.27(85%) 6.84(15%)	3.81	0.75	1.88
**16a**									
c-hex	501.5	4.0	517.0	600	0.39	3.09(95%) 5.61(5%)	3.21	1.21	1.90
Et. Ac.	500.0	4.5	516.0	620	0.31	2.60(64%) 3.82(34%)	2.96	1.04	2.33
MeOH	499.0	3.9	514.5	605	0.35	2.95(90%) 6.03(10%)	3.26	1.07	1.99

Absorption (λ_{ab}) and fluorescence (λ_{fl}) wavelength; molar absorption at the maximum (ε_{max}); Stokes shift ($\Delta\nu_{St}$); fluorescence quantum yield (ϕ); fluorescence lifetime (τ) and amplitude average lifetime $<\tau>$; radiative (k_{fl}) and non-radiative (k_{nr}) rate constants (calculated from the average lifetime).
c-hex: cyclohexane; Et. Ac.: ethyl acetate; MeOH: methanol
